# A Wearable Electrochemical Biosensor for Salivary Detection of Periodontal Inflammation Biomarkers: Molecularly Imprinted Polymer Sensor with Deep Learning Integration

**DOI:** 10.1002/advs.202509658

**Published:** 2025-07-26

**Authors:** Sangheon Jeon, Sung Hyun Kim, Gyeonghwa Heo, Hye Jin Heo, Seon Yeong Chae, Young Woo Kwon, Shin‐Kyu Lee, Dong‐Wook Han, Hyun‐Joo Kim, Yun Hak Kim, Suck Won Hong

**Affiliations:** ^1^ Department of Optics and Mechatronics Engineering Department of Cogno‐Mechatronics Engineering College of Nanoscience and Nanotechnology Pusan National University Busan 46241 Republic of Korea; ^2^ Engineering Research Center for Color‐Modulated Extra‐Sensory Perception Technology Pusan National University Busan 46241 Republic of Korea; ^3^ Department of Anatomy School of Medicine Pusan National University Yangsan 50612 Republic of Korea; ^4^ Medical Research Institute School of Medicine Department of Biomedical Informatics School of Medicine Periodontal Disease Signaling Network Research Center and Dental and Life Science Institute School of Dentistry Pusan National University Yangsan 50612 Republic of Korea; ^5^ Department of Periodontology School of Dentistry Department of Periodontics and Dental Research Institute Pusan National University Dental Hospital Yangsan 50621 Republic of Korea

**Keywords:** electrochemical biosensors, matrix metalloproteinase‐8, molecularly imprinted polymers, point‐of‐care testing, wearable oral healthcare monitoring device

## Abstract

The work presented here introduces a developed electrochemical biosensor for the salivary detection of matrix metalloproteinase‐8 (MMP‐8), utilizing a molecularly imprinted polymer (MIP) matrix based on poly(o‐phenylenediamine). To enhance detection sensitivity and modulate impedance responses, graphene oxide (GO) is incorporated as an interlayer, providing a conductive and chemically stable matrix for precise electrochemical sensing. Density functional theory simulations confirm the formation of highly selective binding sites, further reinforcing the sensor's specificity for MMP‐8 detection. The impedance‐based mechanism allows real‐time, label‐free detection of salivary MMP‐8 by tracking charge transfer resistance changes via the K[Fe(CN)₆]^3^⁻/⁴⁻ redox probe, offering a non‐invasive and highly sensitive alternative to conventional methods. Clinical validation using patient samples demonstrates excellent sensor performance, achieving high specificity and reproducibility. Additionally, a deep learning‐assisted data analysis framework is integrated to enhance diagnostic accuracy by filtering out noise, identifying disease progression trends. Furthermore, a wearable mouthguard platform integrating the MIP‐based electrode, enabling continuous monitoring of oral inflammation and facilitating early therapeutic intervention is developed. This approach, which combines MIP technology, electrochemical biosensing, wearable healthcare, and AI‐driven diagnostics, has the potential to establish a next‐generation precision oral health monitoring platform, advancing periodontal disease detection and personalized clinical management.

## Graphical abstract

A wearable electrochemical biosensor integrated into a smart mouthguard enables the non‐invasive detection of salivary MMP‐8 for early monitoring of periodontal inflammation. Featuring a molecularly imprinted polymer‐based sensing layer, the platform offers high cytokine selectivity and impedance‐driven sensitivity. Combining wireless connectivity with a patient‐friendly design, this compact system provides a robust solution for precision intraoral diagnostics.

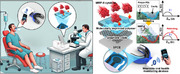

## 1 Introduction

Periodontal diseases, encompassing gingivitis and periodontitis, represent a significant global health burden with profound implications for oral and systemic health.^[^ ^1^, ^2^ ^]^ These inflammatory conditions, driven by oral microbiome dysbiosis, generally lead to the progressive destruction of the periodontium and are increasingly recognized as contributing factors to systemic disease, including diabetes mellitus, cardiovascular disease, and rheumatoid arthritis.^[^ ^3^, ^4^, ^5^ ^]^ Bacterial infections initiate chronic inflammation, which in turn, compromises the structural integrity of the periodontium, expediting the degradation of alveolar bone and periodontal ligaments.^[^ ^6^ ^]^ Substantial evidence suggests that periodontal inflammation extends beyond the oral cavity, exerting systemic effects and establishing strong correlations with metabolic and cardiovascular disorders.^[^ ^7^ ^]^ Despite the established role of clinical assessments, such as probing depth measurements, clinical attachment loss evaluation, and radiographic imaging, in diagnosing periodontal diseases, these methodologies remain inherently limited in their ability to capture dynamic biochemical changes that reflect early disease onset and progression.^[^ ^8^ ^]^ Thus, there is an urgent demand for a non‐invasive, real‐time, and highly sensitive diagnostic tool that enables early detection and continuous monitoring of periodontal inflammation.^[^ ^9^, ^10^ ^]^

Recent advancements in biosensing technologies have facilitated the development of electrochemical sensors based on molecularly imprinted polymer (MIP) technology, a biomimetic strategy that enables highly selective molecular recognition through the formation of artificially engineered receptor sites.^[^ ^11^, ^12^, ^13^ ^]^ Owing to their exceptional chemical stability, reproducibility, and adaptability, MIP‐based electrodes have emerged as promising candidates for wearable biosensing platforms and point‐of‐care (POC) diagnostic devices.^[^ ^14^ ^]^ From a biosensing perspective, MIP‐based electrodes exhibit highly selective molecular recognition, effectively mimicking antibody–antigen interactions while maintaining chemical robustness and structural integrity.^[^ ^15^ ^]^ To enhance the clinical translatability of these sensors, various fabrication strategies have been developed to ensure scalable, cost‐efficient, and reproducible manufacturing, thereby mitigating batch‐to‐batch variability and ensuring consistent sensor performance.^[^ ^16^ ^]^ This is particularly critical for clinical implementation and integration into real‐time biomonitoring platforms, where precision and long‐term stability are paramount. In this context, MIP‐based electrodes offer several distinct advantages, positioning them as next‐generation diagnostic tools.^[^ ^17^ ^]^ Specifically, MIP‐based electrochemical sensors employ an impedance‐based transduction mechanism, enabling real‐time, label‐free monitoring of biomarker interactions and providing a continuous, dynamic assessment of periodontal inflammation.^[^ ^18^ ^]^ Among the various biomarkers implicated in periodontal inflammation, matrix metalloproteinase‐8 (MMP‐8) has been identified as a highly specific indicator due to its direct role in collagen degradation and extracellular matrix remodeling.^[^ ^19^, ^20^, ^21^, ^22^, ^23^, ^24^ ^]^ Elevated salivary and gingival crevicular fluid MMP‐8 levels correlate strongly with active periodontal destruction, making it a compelling target for early disease detection.^[^ ^25^, ^26^ ^]^ However, traditional laboratory‐based MMP‐8 assays, such as enzyme‐linked immunosorbent assays (ELISA) and mass spectrometry (MS), while highly sensitive, suffer from long processing times, dependency on specialized instrumentation, and impracticality for point‐of‐care testing (POCT) applications.^[^ ^27^, ^28^ ^]^ By integrating MMP‐8‐specific recognition elements into MIP‐based electrochemical biosensors, real‐time periodontal inflammation monitoring can be achieved with high specificity, providing a practical and clinically relevant diagnostic approach for early detection and disease progression tracking.

**Scheme 1:**
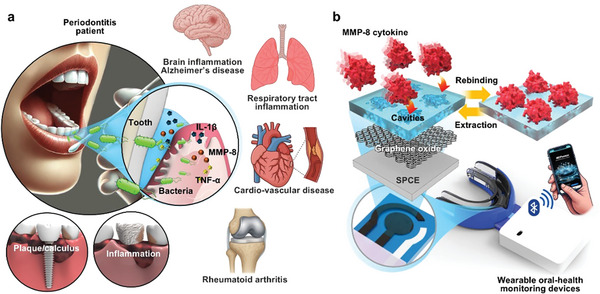
a) Systemic comorbidities associated with periodontal disease, driven by cytokine cascades and pathogenic mechanisms. b) Schematic illustration of the electrochemical detection of MMP‐8 cytokine using an MIP‐based electrode with synthetic receptors (i.e., cavities), designed for integration into a wearable oral health monitoring device.

Herein, we report the development of a wearable electrochemical biosensor for salivary MMP‐8 detection, incorporating an MIP based on poly(o‐phenylenediamine) (poly(o‐PD)) as the molecular recognition matrix. This material system was selected for its robust molecular recognition capabilities and high structural stability, ensuring reliable biomarker detection in complex biological environments.^[^ ^29^, ^30^ ^]^ In the layered electrode architecture, graphene oxide (GO) was strategically incorporated as an interlayer within the MIP‐based bioelectrodes to enhance detection sensitivity and precisely modulate impedance responses. The addition of GO provides a conductive and chemically stable matrix, significantly improving electrochemical detection performance. Furthermore, density functional theory (DFT) simulations were employed to understand the molecular interactions between the MIP matrix (poly(o‐PD)) and the target biomolecule (MMP‐8). These computational analyses supported the optimized molecular imprinting strategy and corroborated the MIP‐based electrode's high specificity for MMP‐8 detection as demonstrated experimentally. At the core of this biosensing platform lies an impedance‐based electrochemical sensing mechanism, which capitalizes on biomarker‐induced perturbations in electrical resistance to enable highly sensitive, label‐free, and real‐time detection. Unlike conventional detection methods,^[^ ^31^ ^]^ this approach provides continuous, non‐invasive monitoring of biomolecular interactions in complex biological matrices, such as saliva, with high analytical specificity and precision. To evaluate the clinical applicability and translational potential of our MIP‐based biosensing platform, we conducted an extensive set of validation studies, incorporating clinical patient samples to assess sensing performance. Additionally, we implemented a deep learning (DL)‐based data processing framework, integrating predictive analytics to enhance diagnostic accuracy and clinical utility.^[^ ^32^, ^33^ ^]^ By leveraging AI‐driven analysis, this system effectively filters out noise, identifies disease progression trends, and provides personalized health insights, further improving the reliability of biosensor readings.^[^ ^34^ ^]^ Beyond the MIP‐based electrode itself, wearable oral health monitoring devices, such as mouthguard, embedded with saliva‐sampling component, hold immense potential as non‐invasive diagnostic platforms. By integrating MIP‐based electrochemical electrodes with these smart oral devices and wireless data transmission systems, we establish a patient‐compliant approach that enables continuous tracking of oral inflammation dynamics and facilitates early therapeutic intervention. Therefore, by bridging the fields of MIP technology, electrochemical biosensing, wearable healthcare, and DL‐enhanced diagnostics, this research aims to establish a next‐generation precision oral health monitoring technology capable of transforming the early detection and management of periodontal diseases (**Scheme** **1**). We believe that our work demonstrates the feasibility of integrating advanced biosensing platforms into routine periodontal disease screening and personalized oral health management, thereby maximizing the diagnostic potential of salivary biomonitoring for future clinical applications.^[^ ^35^ ^]^

## 2 Results and Discussion

### 2.1 Scalable, Reproducible Manufacturing of MIP Electrodes: Utilizing GO Interlayers for Improved Sensitivity and Stability

**Figure 1:**
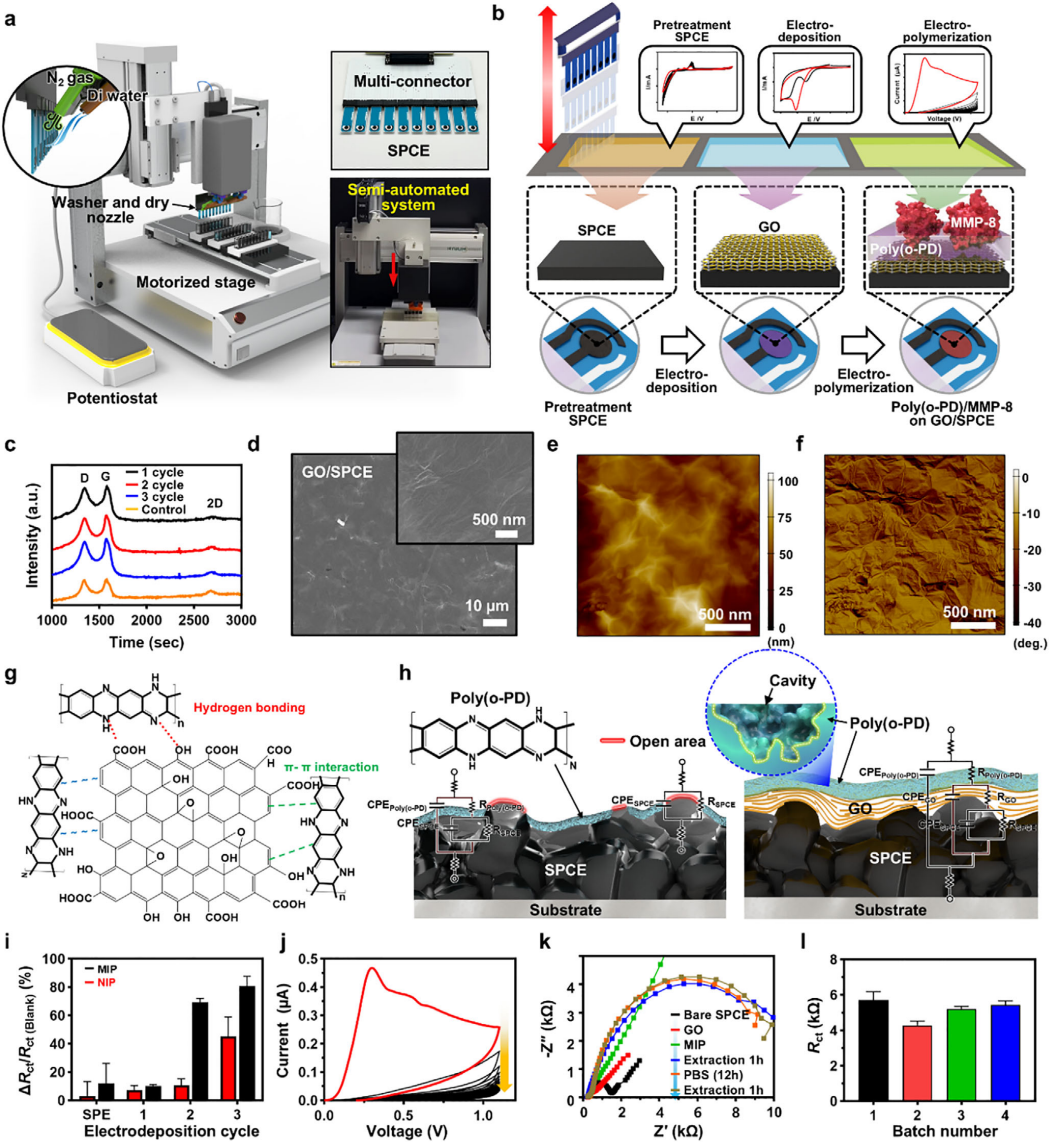
a) Conceptual illustration of the semi‐automated manufacturing system for high‐throughput MIP‐based electrode. b) Stepwise schematic of the fabrication process, including SPCE pretreatment, GO deposition, o‐PD electropolymerization, and template extraction. c) Raman spectra of GO layers across multiple deposition cycles. d) SEM images depicting uniform GO layers on SPCE surfaces. e,f) AFM topographical and phase mapping of the GO layer. g) Conceptual diagram illustrating GO/o‐PD interactions. h) Equivalent circuit model demonstrating GO‐mediated *R*_ct_ modulation and sensor stability enhancement. i) Concentration‐response of MIP and NIP electrodes as a function of GO deposition cycles. j) Electropolymerization curves of o‐PD/MMP‐8 solutions on GO layers. k) EIS response variations across different fabrication stages. l) Batch‐to‐batch reproducibility assessment of MIP electrodes fabricated using the semi‐automated system.

**Figure** **1**a illustrates a semi‐automated system designed for the fabrication of MIP‐based electrodes. Traditional MIP fabrication methods rely heavily on careful manual electrochemical deposition with limitations in scalability and reproducibility.^[^ ^36^, ^37^ ^]^ To improve these, our semi‐automated system allows continuous processing with real‐time input of materials and output of MIP‐functionalized electrodes for data analysis, reducing manufacturing time by up to ≈90% with improved batch‐to‐batch consistency. For example, it produces 30 samples in just 2 h compared to 25 h manually, offering an economical advantage for the sample preparation. In the manufacturing system, key components include a potentiostat, 3‐axis motorized stage, programmable user‐interface, innovative connectors, solution cuvettes, and cleaning/drying units, enabling semi‐automated 15‐min process intervals, as demonstrated in Movie S1 (Supporting Information). Controlled via a G‐code‐based program with an intuitive user interface, the system achieves medium automation (e.g., Level 3, ISO 10218‐1). Notably, its engineered multi‐terminal connectors, combined with a symmetrically designed circuit layout, ensured uniform current application across 10 screen‐printed carbon electrodes (SPCEs), thereby enhancing reproducibility and signal consistency, as validated by quantitative structural design analysis (Figure S1, Supporting Information). During sample production, the potentiostat simultaneously applied identical voltage and current levels through these multi‐terminal connectors, maintaining stable electrochemical reactions throughout pretreatment, GO interlayer deposition, and polymerization steps across all electrodes (Figure S2, Supporting Information).

Figure 1b outlines the detailed processing steps of the semi‐automated manufacturing process, divided into three major stages that contribute to electrode functionality. The process began with the pretreatment of SPCEs using sulfuric acid (H₂SO₄) to remove surface impurities and activate the electrode. Subsequently, GO was electrochemically deposited onto the SPCE surface. The high surface area and abundant oxygen‐containing functional groups of GO facilitate strong chemical interactions with o‐PD monomers during electrochemical polymerization, thereby improving the formation and sensitivity of the molecular recognition layer. Through this approach, a stable polymeric matrix can be formed on the electrode surface by incorporating protein templates with monomers. Finally, template proteins were extracted using a 1:1 methanol and DI water solution (Figure S3, Supporting Information), creating imprinted cavities on the SPCE surface for selective target protein recognition. Schematics in the lower panels of Figure 1b depict the layered film surfaces at each step, illustrating cavity formation critical to detection performance. In this experimental concept, the GO film plays a pivotal role as an interlayer in the MIP structure, enhancing molecular sensing properties. As noted, the functional groups in GO, such as epoxides, hydroxyls, and carboxyls, aid dispersion in aqueous solution but disrupt sp^2^ hybridized orbitals, reducing conductivity.^[^ ^38^, ^39^ ^]^ Thus, partial electrochemical reduction of GO can be an effective strategy for tuning its electrochemical performance by slightly restoring conductivity while retaining functional groups. As evidenced by monitoring electrochemical deposition steps, the electrochemical reduction of GO was detected by distinct current–voltage profiles (Figure S4, Supporting Information). In the initial reduction step, a marked cathodic peak at −0.7 V indicated oxygen group removal, with subsequent scans showing increased peak currents from the formation of a conductive layer. Obviously, electrochemical impedance spectroscopy (EIS) and cyclic voltammetry (CV) analyses confirmed enhanced impedance responses after GO film deposition, as presented in Figure S5a–c (Supporting Information). Raman spectroscopy also demonstrated structural changes in the GO film, with the *I*_D_/*I*_G_ ratio decreasing from 0.95 to 0.93 following partial reduction, which reflects a balance between maintaining functional groups and restoring conductivity (Figure 1c), as changes in the D and G bands indicated defect evolution and sp^2^ carbon domain restoration.^[^ ^40^, ^41^ ^]^ As shown in Figure 1d, the morphological features of the GO film on the SPCE surface, under optimized conditions (Figure S6, Supporting Information), were measured by scanning electron microscopy (SEM), representing a flattened surface. Tuning the deposition of the GO interlayer was essential for optimal performance; excessive thickness increases insulation and reduces conductivity, while insufficient thickness leads to uneven MIP film deposition. Complementary atomic force microscopy (AFM) measurements (Figure 1e,f) confirmed the formation of highly uniform GO layers deposited onto the SPCE surface, exhibiting a significantly reduced root‐mean‐square (RMS) roughness of ≈150 nm, compared to ≈314 nm for the bare SPCE, based on 10 × 10 µm^2^ scan areas (Figure S7, Supporting Information). Additionally, cross‐sectional analysis (Figure S8, Supporting Information) further demonstrates controlled modulation of GO interlayer thickness and homogeneity, which facilitates uniform MIP film formation and contributes to enhanced sensing performance.

From the perspective of surface chemistry, Figure 1g illustrates the coupling role of GO during the polymerization of o‐PD. The functional groups of GO facilitate intimate molecular interactions with o‐PD monomers through hydrogen bonding, *π–π* interactions, and van der Waals forces, which promote uniform monomer adsorption and enhance stability of the MIP matrix. Unlike previous studies that rely on concentration‐response correlations without exploring intermediate layer interactions,^[^ ^42^, ^43^ ^]^ our work advances the understanding of the MIP‐based sensing mechanism by investigating the specific role of the interlayer (i.e., GO film) by establishing a tunable correlation between its electrochemical properties and sensor performance. To scrutinize the GO interface and its associated electrochemical behaviors, Figure 1h presents an equivalent circuit model, incorporating a constant phase element (CPE) to consider the capacitive effects of the poly(o‐PD)‐based MIP/GO films and the charge transfer resistance (*R*_ct_) to modulate subtle interfacial parameters. Here, we hypothesize that the electrochemically deposited GO interlayer on the SPCE serves as a charge transfer stabilizing element for *R*_ct_ values to guarantee reproducible sensor performance. Functioning as a physical barrier and an electrochemical mediator, the GO interlayer is designed to enhance the structural reliability of the overlying MIP, which exhibits high coverage of molecular cavities (i.e., rebinding sites) after MMP‐8 template extraction, thereby enabling selective recognition of target molecules.

Figure 1i summarizes the impact on the GO deposition cycles with sensing performance. Normalized Δ*R*_ct_/*R*_ct (Blank)_ values were extracted from the EIS responses as a function of GO deposition cycles to assess the electrochemical effect of the GO interlayer in the MIP structure, which was evaluated within a certain MMP‐8 concentration range (i.e., 200 ng mL⁻¹). For example, after a single deposition cycle, incomplete coverage of the SPCE surface by the GO interlayer resulted in partially exposed electrode regions, leading to similar electrochemical signals in the comparable response range between MIP and NIP (non‐imprinted polymer) electrodes. In contrast, a 2‐cycle deposition facilitated the formation of a more uniform GO interlayer, yielding well‐defined stable sensing responses with distinct relative *R*_ct_ value differences and enhanced sensitivity. However, a 3‐cycle deposition caused a substantial increase in *R*_ct_ due to the insulating properties of the thicker GO layer, which impeded electron transfer. As a result, the sensitivity difference between MIP and NIP electrodes was notably reduced compared to that observed in the samples from 2‐cycle deposition. Thus, as previously discussed, the optimized GO interlayer formation on the SPCE is an important factor in balancing the CPE circuit configuration by modulating charge transfer and interfacial conductivity. Experimental observations indicate that the optimal GO interlayer thickness was ≈20–30 nm, achieved through 2‐cycle electrodeposition, maintaining consistent impedimetric responses.

Simultaneously with the optimization of the GO interlayer functionality, sequential measurements of the electrochemical polymerization of o‐PD exhibited a progressive decrease in oxidation peak current over successive scans (Figure 1j). This trend confirms the gradual formation of the poly(o‐PD) matrix, driven by enhanced interactions between GO and the o‐PD monomer.^[^ ^44^ ^]^ Notably, the formation of the poly(o‐PD) film was verified through optical imaging and direct visual inspection (Figure S9, Supporting Information), further attesting to the uniform synthesis of the MIP film on the GO surface. Additional EIS and CV analyses (Figure 1k; Figure S10, Supporting Information) captured the evolving electrochemical characteristics at each stage of synthesis. In particular, following template removal from the poly(o‐PD) matrix, distinct impedance features were observed as ion pathways were re‐established under controlled polymerization conditions, forming functional sensing layers. Consistent sensor performance was evidenced by comparable variations in initial *R*_ct_ values (Figure 1l). Importantly, our manufacturing system exhibited high reproducibility and stability, with a relative standard deviation (RSD) of ≈4.5% across production batches using semi‐automated equipment. Moreover, the minimal *R*_ct_ variation between batches (RSD ≈3.7%) showed the reliability of our fabrication conditions, supporting scalability for high‐throughput MIP‐based electrode production.

### 2.2 Integration of Computational and Spectroscopic Techniques for MIP Optimization

**Figure 2:**
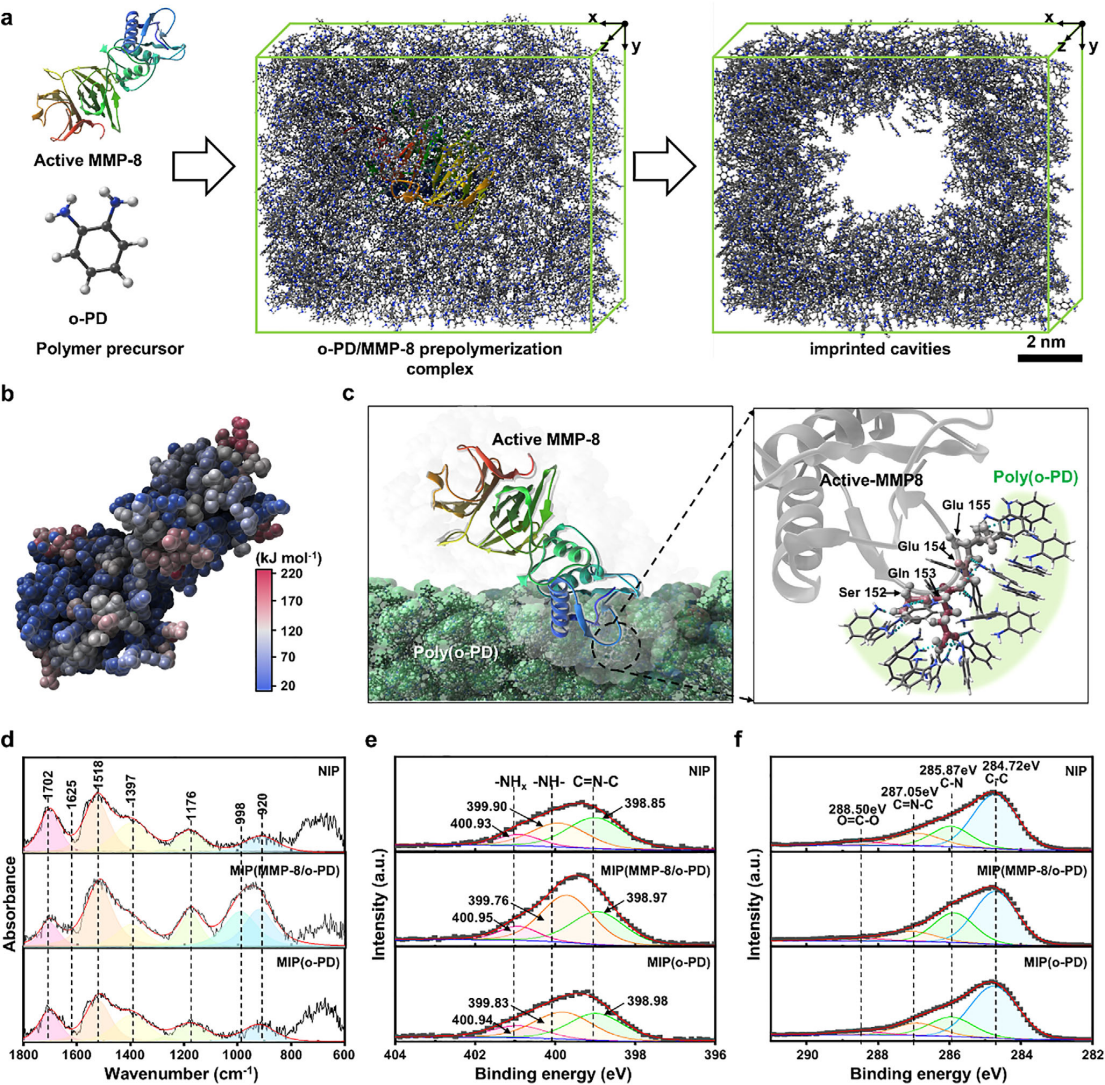
Computational and experimental validation of MMP‐8 imprinting specificity. a) MD simulation schematic illustrating the formation of the o‐PD/MMP‐8 prepolymerization complex and the predicted morphology of imprinted cavities. b) Binding energy mapping of o‐PD monomers on the MMP‐8 surface, highlighting key epitopes for selective imprinting. c) Paratope‐epitope interactions between poly(o‐PD) and MMP‐8, emphasizing the specific amino acid sequences involved in binding. d) FT‐ATR spectra of MIP and NIP films, confirming the retention of functional groups after template extraction. e,f) XPS analysis of the N 1s and C 1s regions, providing the chemical composition and binding characteristics of the MIP surface.

The specific binding of MMP‐8 protein within the poly(o‐PD) matrix is a critical determinant of sensor performance.^[^ ^45^ ^]^ To enable accurate quantitative prediction, we conducted preliminary investigations using protein‐ligand molecular dynamics (MD) simulations and density functional theory (DFT) calculations.^[^ ^46^, ^47^, ^48^, ^49^ ^]^ **Figure** **2**a presents the MD simulation results, providing structural insights into the prepolymerization complex formed between o‐PD monomer and active MMP‐8. The top‐left panel of Figure 2a displays a ribbon diagram representation of the active MMP‐8 protein, modeled to mimic its physiological conformation in saliva during periodontitis. The molecular bond angles and folding structure of the protein were derived from AlphaFold deep learning model predictions (UniProt: P22894) and used as foundational data for subsequent MD simulations.^[^ ^50^ ^]^ Structurally, the MMP‐8 protein consists of an N‐terminal sequence (residues 1–20), a propeptide domain (residues 1–108) responsible for peptidoglycan binding, a catalytic domain (residues 109–275) functioning as a zinc‐dependent metalloprotease, and a hemopexin‐like domain (residues 276–467) formed by four β‐sheets. Given that the propeptide domain is cleaved during enzymatic activation and lacks specificity in biosensing applications, it was excluded from this study. Instead, we focused on the catalytic and hemopexin‐like domains, which are connected by a flexible hinge region and contribute significantly to the binding specificity of MIP‐based sensing electrodes. However, due to the inherent conformational flexibility of these domains, simple physical imprinting alone is insufficient to induce the specific chemical interactions necessary for high‐performance biosensing. Consequently, identifying the precise protein sequences that function as epitopes, akin to antigen–antibody interactions, is essential for achieving targeted detection of MMP‐8 and optimizing MIP‐functionalized electrode design.^[^ ^49^ ^]^ The bottom‐left panel of Figure 2a illustrates the molecular structure of o‐PD, which was derived using the semi‐empirical parameterization method, referencing values from a DFT simulation library. The central panel presents the MD simulation results, where 2500 o‐PD monomers were randomly distributed around active MMP‐8 within a 10 nm × 10 nm × 10 nm simulation box, followed by energy minimization. The right panel depicts the predicted 3D morphology of the polymer matrix following MMP‐8 extraction, revealing an imprint cavity designed for highly selective molecular recognition. Supporting evidence in Figure S11 (Supporting Information) further demonstrates that the prepolymerization complex of o‐PD and active MMP‐8 forms an ellipsoidal cavity measuring ≈9 nm × 4 nm × 4 nm.

A key finding from the MD simulations was the non‐uniform distribution of hydrogen bonds between the 358 amino acid residues of MMP‐8 (residues 109–467) and ≈490 o‐PD molecules. This distribution was strongly influenced by the electrostatic partial charges on the MMP‐8 surface and steric hindrance arising from internal protein sequences.^[^ ^51^ ^]^ Using the Hydrogen Bond Finder tool in SAMSON molecular modeling software, we analyzed the polarity of interatomic interactions (e.g., O─H, C═O, and N─H) and their intermolecular distances. By integrating these findings with DFT calculations, we elucidated the non‐covalent binding interface between MMP‐8 and prepolymerized o‐PD, allowing for a quantitative estimation of binding energy. Consistent with prior studies,^[^ ^52^ ^]^ the electrostatic potential (ESP) of active MMP‐8 protein sequences was thoroughly computed. The non‐covalent binding energy of the prepolymerization complex was further estimated using the polarized continuum model (Binding energy (*ΔE*) = *E_complex_* – *E_o‐PD_* – *E_amino acid_*), revealing insights into the fundamental molecular interactions governing MMP‐8 recognition within the MIP structure. Table S1 (Supporting Information) presents the binding energies of 20 amino acids interacting with o‐PD monomers, as computed via DFT simulations using ESP analysis. Figure 2b maps these binding energies onto the van der Waals surface of the active MMP‐8 protein, providing a spatial visualization of the relative binding strengths of o‐PD to specific protein sequences. This mapping integrates the predicted hydrogen bonding interactions obtained from MD simulations, offering comprehensive insight into the imprinting process. Further illustrating these interactions in Movie S2 (Supporting Information) provides a rotational visualization of the active MMP‐8 van der Waals surface, accentuating localized regions with particularly high binding energies. For benchmarking, Table S2 (Supporting Information) compares the binding interaction profiles of o‐PD and eriochrome black T (EBT) monomers with MMP‐8 epitopes. Although EBT was successfully employed in prior work for IL‐1β detection via EBT‐based MIP systems,^[^ ^16^ ^]^ its prepolymerization interaction modeling with MMP‐8 exhibited more dispersed and less focused binding energy distributions. In contrast, o‐PD demonstrated more spatially localized and energetically concentrated interactions with MMP‐8 epitopes, supporting its superior imprint specificity for this target. Notably, while the o‐PD system exhibited ≈14.3% lower average binding energies compared to EBT, it showed a ≈9.8% higher RSD in binding energy distribution, indicating that the binding events were more localized rather than randomly dispersed. This concentrated interaction pattern is critical for forming selective binding sites, thereby enhancing the molecular recognition capabilities and suggesting an enhancement in sensor performance due to improved imprint specificity. As depicted in the Movie S2 (Supporting Information), the van der Waals surface of the prepolymerized o‐PD/active MMP‐8 complex reveals concentrated high‐affinity binding sites (highlighted in red). The pronounced ESP differences in the o‐PD system facilitated the precise alignment of coordinating o‐PD molecules, increasing the capability of polymer formation while preserving the geometric integrity of the prepolymerization complex. These findings collectively validate the superior molecular recognition capability of the o‐PD‐based MIP system.

Figure 2c illustrates the formation of paratopes on the poly(o‐PD) surface through electropolymerization. The corresponding epitope sequences within the MMP‐8 protein serve as critical structural components, governing the specificity of the MIP‐functionalized electrode. The magnified panel in Figure 2c further emphasizes key amino acid residues, such as Ser 152, Gln 153, Gly 154, and Glu 155 that participate in binding interactions with the poly(o‐PD) paratopes. Additionally, Figure S12 (Supporting Information) provides further visualizations of hydrogen bond formation within the prepolymerization complex, along with estimated binding energies for various epitopes. For instance, the binding energy of epitope 152–155 was determined to be ≈697 kJ mol⁻¹ (averaging ≈43.5 kJ mol⁻¹ per atom, excluding hydrogen), indicative of strong non‐covalent interactions. Other notable epitopes exhibiting significant binding affinities included sequences 115–120, 164–169, 205–210, and 392–403, with average binding energies ranging from ≈37.9 to ≈41.1 kJ mol⁻¹ per atom. However, it is important to recognize that not all epitopes can simultaneously engage in binding interactions, contributing to variability in overall MIP binding energy.^[^ ^53^ ^]^ The structural flexibility of the hemopexin‐like and catalytic domains suggests that excessive MIP film thickness may hinder specific binding, thereby reducing imprint specificity. These results represent the critical role of controlled MIP synthesis in ensuring optimal sensing performance. By integrating MD simulations with DFT calculations, we predicted that the prepolymerization complex of o‐PD/active MMP‐8 would retain selective and specific binding properties post‐electropolymerization. This study provides a comprehensive mechanistic understanding of the interactions between poly(o‐PD) and active MMP‐8, demonstrating their potential to facilitate high‐selectivity imprinting for the advanced design of the developed MIP‐functionalized electrode.

To further substantiate these findings derived from MD and DFT simulations, spectroscopic surface analyses were conducted to examine the chemical functionalities present on the MIP‐functionalized electrode surface. Figure 2d presents the results of Fourier transform attenuated total reflection (FT‐ATR) spectroscopy, performed on various samples: NIP (non‐imprinted polymer) fabricated without the MMP‐8 template, MIP synthesized with MMP‐8, and MIP following MMP‐8 extraction. Across all samples, seven characteristic molecular vibration modes were identified, as summarized in Table S3 (Supporting Information). These included vibrations at 1702 cm⁻¹ (C═N stretching), 1518 cm⁻¹ (aromatic carbon), and 1176 cm⁻¹ (C─N stretching), all intrinsic to poly(o‐PD).^[^ ^54^, ^55^ ^]^ Notably, the absence of significant spectral shifts before and after MMP‐8 extraction suggests that the poly(o‐PD)‐based MIP layer remained structurally intact throughout the process. The vibration modes at 1397 and 920 cm⁻¹, attributed to C─H stretching, were present in both poly(o‐PD) and MMP‐8, complicating the quantitative interpretation of these peaks. However, vibrations at 998 and 920 cm⁻¹, associated with ─C─OH═C bonds, were unique to amino acid sequences and displayed distinct spectral variations depending on MMP‐8 extraction, reinforcing the effectiveness of the imprinting process. The spectral region between 800 and 600 cm⁻¹, corresponding to N─H bending modes (wagging and rocking asymmetrical stretching), remained unresolved, likely due to peak overlap and limited resolution.^[^ ^56^, ^57^ ^]^

To cross‐validate the FT‐ATR findings, X‐ray photoelectron spectroscopy (XPS) analyses were performed on identically prepared samples. Figure 2e displays the high‐resolution N 1s XPS spectrum, revealing distinct peaks at 400.93 eV (─NH_x_─), 399.76 eV (─NH─), and 398.85 eV (C═N─C), corresponding to chemical bonds in both poly(o‐PD) and MMP‐8.^[^ ^58^ ^]^ However, in the MIP sample containing MMP‐8, which incorporated a surface‐imprinted MMP‐8 layer, a significantly higher fraction of peptide bonds (C═O─NH─) was observed, exhibiting a binding energy shift of ≈0.4 eV due to shielding effects. Additionally, the quantitative area ratio of the ─NH─peak increased by ≈35.7%, consistent with the FT‐ATR results. Figure 2f illustrates the high‐resolution C 1s XPS spectrum, where peaks were assigned to 287.05 eV (C═N─C) and 284.72 eV (C─C) for the poly(o‐PD) MIP layer, along with 285.87 eV (C─N) for defect sites in poly(o‐PD) or amino acid residues and 288.50 eV (O═C─O) for non‐specifically adsorbed carbon species.^[^ ^59^, ^60^ ^]^ The MIP sample with MMP‐8 template exhibited a ≈19.8% increase in the C─N peak, indicating distinct modifications in surface chemical bonding due to the presence of amino acids. Based on the integration of MD simulations, DFT calculations, and spectroscopic surface energy analyses, the poly(o‐PD) and MMP‐8 imprinting system developed in this study provides an optimized biorecognition strategy with a well‐defined sensing mechanism. This system demonstrates superior selectivity and sensitivity compared to previously reported MIP platforms, attributed to the precisely engineered molecular imprinting parameters tailored for MMP‐8 cavity formation. The collective results confirm that the MIP electrode successfully achieves high selectivity, ensuring robust performance for precise biomolecular detection.

### 2.3 Operational Principle and Performance of the MIP‐Based Electrochemical Electrode

**Figure 3:**
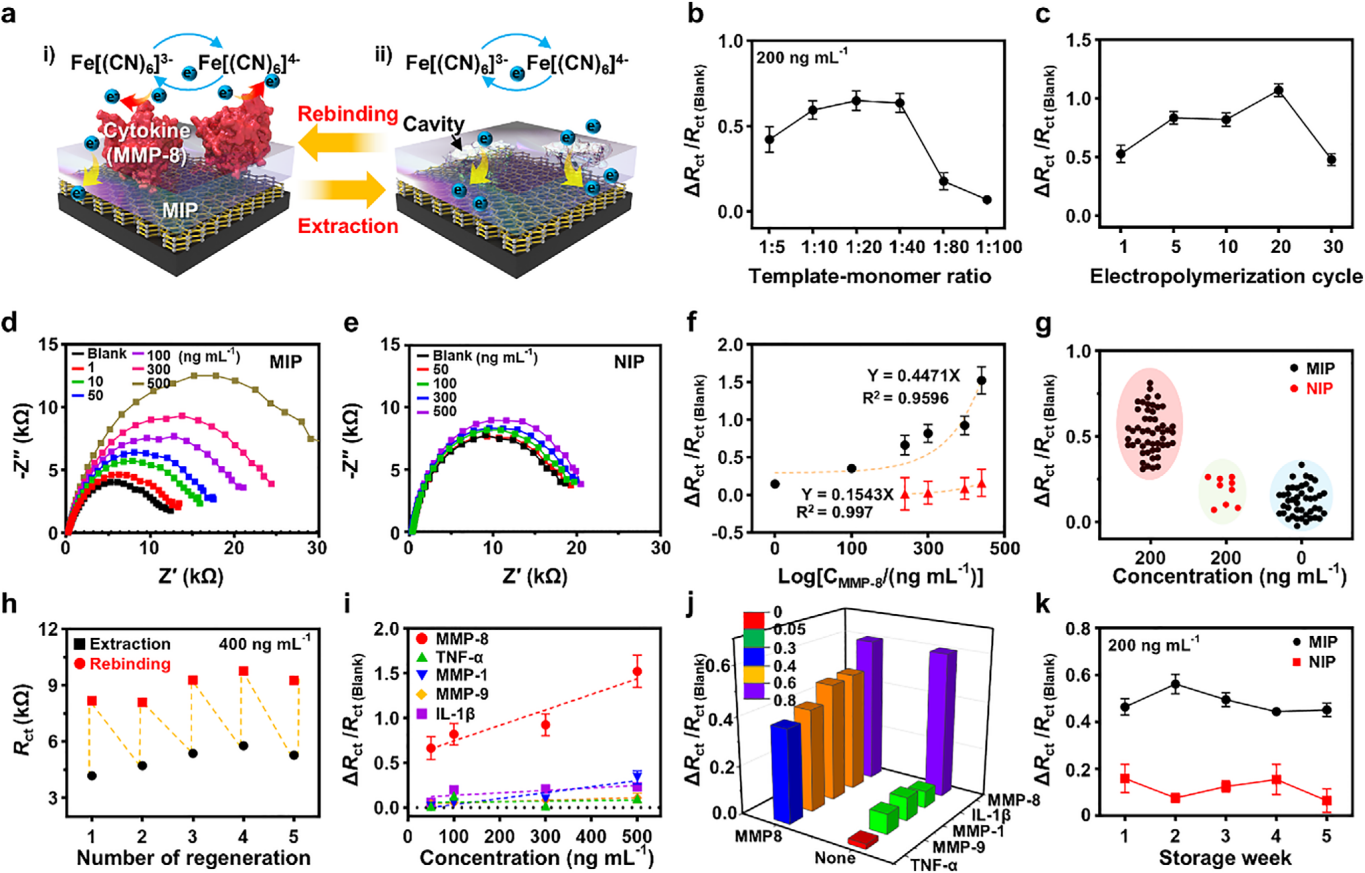
Performance evaluation of MIP‐based electrodes for MMP‐8 detection. a) Schematic representation of the MIP electrode‐based sensor operation, illustrating selective MMP‐8 recognition and redox probe‐mediated signal transduction. b,c) EIS response data for monomer‐to‐template ratio optimization and electropolymerization cycle variation, respectively, to maximize binding efficiency. d,e) EIS responses of MIP and NIP electrodes across various MMP‐8 concentrations. f) Calibration curve of the sensor, demonstrating sensitivity and detection limits. g) Reproducibility assessment of 100‐sensor repetitions, ensuring consistent performance. h) Reusability evaluation of the MIP electrode, maintaining >90% response up to 5 detection cycles. i) Selectivity analysis against interferents, including TNF‐α, MMP‐1, MMP‐9, and IL‐1β. j) Interference analysis using binary mixtures of MMP‐8 with each interferent. k) Long‐term stability evaluation of the MIP electrodes under nitrogen storage conditions.

**Figure** **3**a illustrates the functional mechanism of the MIP‐based electrochemical electrode, in which a surface‐immobilized molecular imprinting system facilitates the selective recognition of MMP‐8 cytokine. This architecture enables specific rebinding interactions between the analyte and the preformed molecular cavities, ensuring highly accurate quantitative detection. As depicted in the left panel, molecularly imprinted cavities are generated by incorporating the target protein into the MIP matrix, followed by template extraction (right panel). This process grants the MIP‐based electrode exceptional molecular recognition capability and specificity, functioning as an artificial receptor for MMP‐8. The sensing mechanism operates by introducing a redox probe (K₃[Fe(CN)₆]/K₄[Fe(CN)₆]) onto the electrode surface, where the presence of MMP‐8 selectively impedes charge transfer at the electrode/electrolyte interface, leading to measurable electrochemical impedance variations. The study highlights the critical role of surface imprinting techniques in optimizing signal transduction, facilitating efficient mass transfer kinetics, and enhancing rebinding accuracy under experimental conditions. Figure 3b demonstrates that a monomer‐to‐template ratio of 1:40 000 provides optimal binding efficiency for MMP‐8 detection. Increasing this ratio to 1:80 000 reduces template availability during electropolymerization, impairing cavity formation and diminishing sensing electrode performance. Similarly, Figure 3c reveals that sensing efficiency peaks at 20 polymerization cycles, balancing polymer thickness and cavity accessibility. Beyond 30‐cycles, excessive polymer deposition results in partial template entrapment within the poly(o‐PD) matrix, thereby reducing MMP‐8 capturing capability. Furthermore, buffer pH critically influences sensing performance by modulating electrostatic interactions, ionic strength, and polymerization dynamics. The highest selectivity and sensitivity were achieved at pH 7.4 (Figure S13, Supporting Information),^[^ ^61^, ^62^, ^63^ ^]^ despite theoretical advantages of surface‐assisted binding (SAB, pH 4.5) conditions for enhancing electrostatic attraction between MMP‐8 (pI: 6.45) and the negatively charged GO/SPCE surface.^[^ ^64^ ^]^ These findings highlight the synergistic role of pH in molecular recognition and polymer formation.

Figure 3d presents the impedance‐based, concentration‐dependent response of the MIP‐functionalized electrode to MMP‐8 cytokine within a range of 1–500 ng mL⁻¹. EIS was performed with the analyte solutions retained on each electrode surface throughout the measurement process. The frequency range most relevant to evaluating the characteristics of the MIP electrode is between 100 kHz and 0.05 Hz, which is visually highlighted in Figure 3d,e. As measured separately across a range of MMP‐8 concentrations, the Nyquist plot exhibited a progressive increase in semicircle diameters (i.e., *R*_ct_) with increasing MMP‐8 concentrations, confirming that analyte rebinding within the imprinted cavities hindered redox probe diffusion at the electrode/electrolyte interface.^[^ ^65^ ^]^ In contrast, NIP electrodes displayed negligible *R*_ct_ variations, reaffirming the high specificity of the MIP‐based electrode (Figure 3e). Figure 3f quantitatively demonstrates a linear correlation between *R*_ct_ values and MMP‐8 concentrations for MIP‐functionalized electrodes, whereas NIP electrodes showed no concentration‐dependent response, further validating the fidelity of molecular imprinting. The limit of detection (LOD) and limit of quantification (LOQ) were calculated using the k × S m^−1^ formula, where S represents the *y*‐intercept, m denotes sensor sensitivity, and k corresponds to the signal‐to‐noise ratio, yielding values of ≈82 ng mL⁻¹ (LOD) and ≈250 ng mL⁻¹ (LOQ). These findings confirm the high sensitivity of electropolymerized MIP films on SPCEs, enabling selective detection of MMP‐8 across a broad dynamic range. To assess the sensor's reproducibility, Figure 3g presents impedance responses obtained from 100 sensor replicates, demonstrating stable baseline signals (i.e., analyte‐free buffer solution) and robust, reproducible impedance shifts at 200 ng mL⁻¹ MMP‐8. The sensors exhibited a reproducibility rate exceeding 90%, reinforcing its potential for clinical diagnostic applications and its suitability for POC biomarker detection.

As illustrated in Figure 3h, the reusability of the MIP‐based electrodes was evaluated by repeatedly extracting and rebinding MMP‐8 across multiple test cycles. Notably, the electrode retained ≈90% of its initial response even after five rebinding cycles, demonstrating stable and reproducible performance. This stability is attributed to the strong interfacial adhesion between the SPCE surface and the electrochemically deposited GO layer, which facilitates robust MIP film formation and preserves cavity integrity during repeated extraction steps (Experimental Section). This highlights the effectiveness of the molecular surface imprinting strategy, demonstrating that well‐defined cavities contribute to the sustained durability and structural integrity of the MIP sensing layer. Furthermore, to assess the specific binding capability of the MMP‐8‐imprinted MIP film, selectivity tests were conducted using various protein species, both individually and in mixtures. Candidate interfering proteins were carefully selected based on molecular weight and isoelectric point (pI) similarity to MMP‐8, simulating potential cytokine interactions within saliva. In the context of molecular sensing electrode performance, it is essential to consider the electrostatic properties of proteins, particularly how solution pH influences their net charge, as changes in charge state can modulate molecular interactions and alter binding affinity at the electrodes’ surface. To investigate the molecular specificity of the MIP electrode over a physiologically relevant concentration range (50–500 ng mL⁻¹ in PBS), the binding characteristic of MMP‐8 (MW: 52.6 kDa; pI: 6.45) was systematically compared against structurally or functionally related cytokines, including TNF‐α (MW: 17.5 kDa; pI: 5.5), MMP‐1 (MW: 45 kDa; pI: 5.4), MMP‐9 (MW: 77.1 kDa; pI: 4), and IL‐1β (MW: 17 kDa; pI: 5.88). As shown in Figure 3i, experimental results revealed a linear increase in complementary rebinding for MMP‐8, whereas relatively negligible *R*_ct_ variations were observed for the individual interfering proteins. As further illustrated in Figure S14, Supporting Information, the MMP‐8 sensing electrode consistently responded significantly higher Δ*R*_ct_/*R*_ct0_ values compared to the other tested interferents across all concentrations, demonstrating high target specificity. The relative signal contributions were quantified as ≈6.5% (TNF‐α), ≈22.1% (MMP‐1), ≈7.1% (MMP‐9), and ≈16.2% (IL‐1β) with statistical significance (*p* < 0.001, *n *≥ 3). Additionally, as shown in Figure 3j and Figure S15, Supporting Information, a binary mixture of MMP‐8 and TNF‐α resulted in moderate signal suppression (≈22%) due to partial rebinding competition within the MIP‐imprinted electrode, whereas minimal interference was detected when MMP‐8 was mixed with MMP‐1, MMP‐9 or IL‐1β, confirming the MIP electrode's selective detection capabilities. Additionally, to ensure long‐term stability, the MIP‐based electrode was stored under N₂ gas packaging for 6 months, during which signal retention remained >90% at the same analyte concentration level (200 ng mL^−1^). These findings indicate that N₂ gas‐based storage offers a chemically inert environment that preserves the structural fidelity of the sensing interface by displacing reactive species such as oxygen and moisture. The absence of these interfering elements minimizes the degradation of functional sites within the MIP layer, thereby preserving its molecular recognition capacity over extended periods. Drawing a parallel to controlled‐atmosphere packaging used in perishable materials, the N₂ gas atmosphere probably suppresses internal moisture accumulation within the MIP matrix, maintaining the physicochemical stability required for consistent performance (Figure S16, Supporting Information). Comprehensively, the molecular entrapment capacity within MIP‐imprinted cavities exhibited acceptable limitations, but the overall results demonstrate the effectiveness of the MIP approach in selectively detecting MMP‐8 biomarkers in parametric experimental conditions, even in the presence of interfering proteins.

### 2.4 POCT and Wearable MIP‐Based Electrochemical Biosensor Platforms for Salivary MMP‐8 Detection

**Figure 4:**
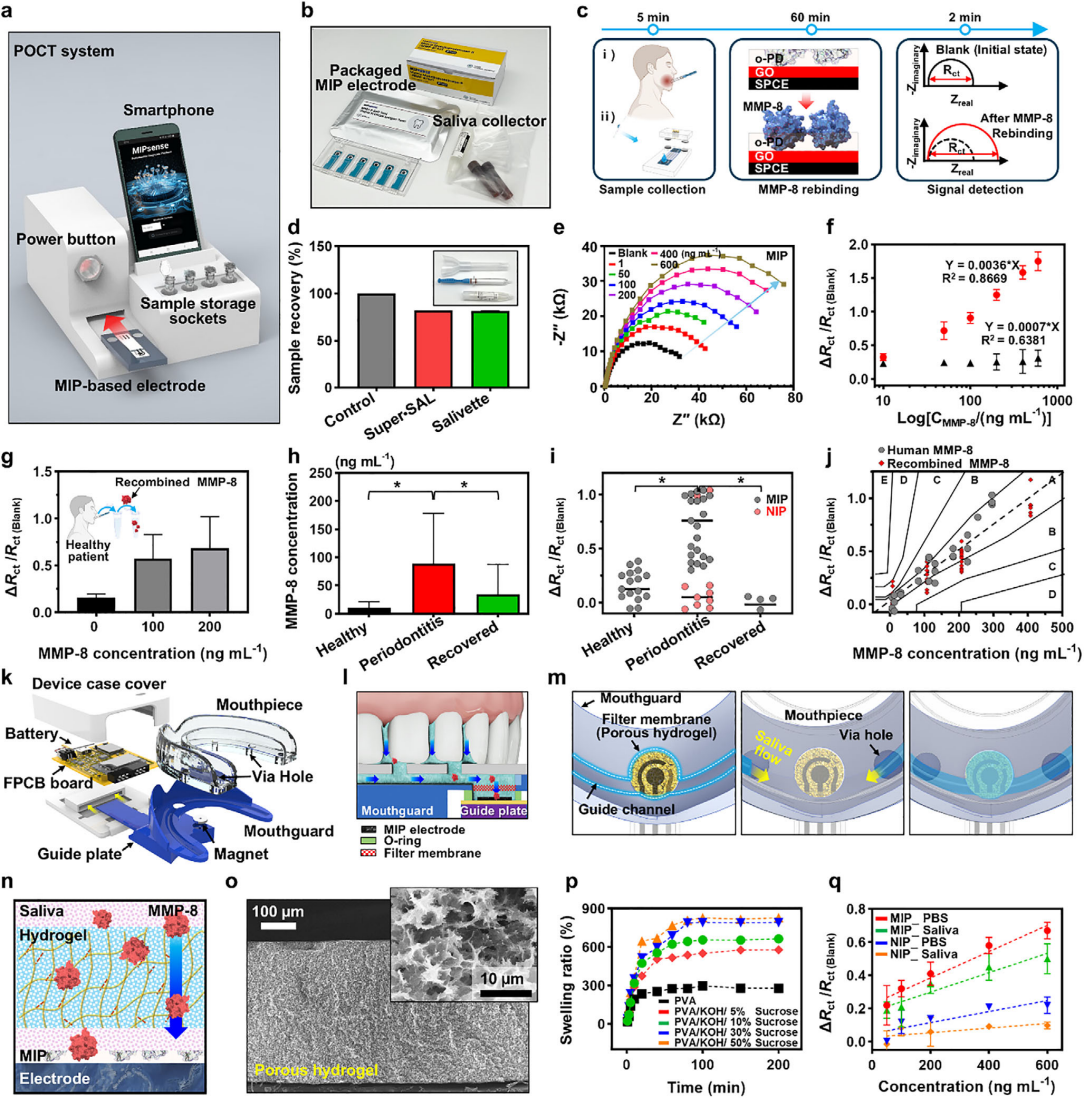
Integrated POCT platform for periodontitis diagnosis and clinical validation. a) Actual image of the MIP‐based POCT prototype incorporating a wireless‐enabled interface. b) Components of the diagnostic kit. c) Schematic workflow of saliva‐based MMP‐8 detection. d) ELISA‐based evaluation of MMP‐8 recovery rates using different pretreatment filters. e) EIS impedance response assessing MIP sensor performance across MMP‐8 concentrations. f) Calibration curves of EIS responses in artificial saliva. g) EIS response of MIP electrode in healthy saliva spiked with MMP‐8. h) ELISA‐quantified MMP‐8 concentrations in clinical cohorts. i) Distribution of EIS responses in ELISA‐validated patient saliva samples. j) Parkes error grid analysis correlating sensor performance with ELISA measurements. k) Modular assembly wearable oral‐health device featuring a mouthpiece/mouthguard with ergonomic design. l) Conceptual schematic of a microfluidic network when worked in the mouth. m) Top‐view of microfluidic network, demonstrating fluidic continuity. n) An illustration of MMP‐8 permeation and contaminant filtration using a PVA‐hydrogel membrane. o) Cross‐sectional SEM image of a freeze‐dried PVA‐hydrogel membrane, confirming uniform porous architecture (inset). p) Swelling ratio analysis of PVA membranes under different fabrication conditions. q) EIS response evaluation of MIP‐based sensors following PVA filtration. Statistical significance is indicated as follows (*n* ≥ 3): *p < 0.05.

**Figures** **4**a and S17 (Supporting Information) illustrate a smart diagnostic POCT platform, featuring a compact sensor unit connected to a smartphone for real‐time signal acquisition and data analysis. This system integrates an MIP‐based electrode, sample collection tools, and electrolyte solutions to facilitate MMP‐8 detection in biological fluids. The user‐centric design prioritizes operational simplicity and portability, enabling standalone analysis without the need for additional equipment. A built‐in potentiostat integrated on a circuit board facilitates precise electrochemical measurements, and a wireless interface allows real‐time data transmission to a dedicated mobile application, configuring sample information and measurement parameters (Figure S18, Movie S3, Supporting Information). Upon sample input, the embedded algorithm interprets electrochemical measurements by analyzing concentration‐dependent impedance inflection points, generating real‐time diagnostic outputs. The sensor's electrical interface utilizes a novel guide‐sliding magnetic interconnection, coupled with a pogo‐pin connector, ensuring secure MIP electrode attachment, minimizing handling errors, and undesired electrode damage. Figure 4b displays the diagnostic kit components, which include a swab‐based pretreatment filter for saliva collection, MIP electrode, an electrolyte solution, and sterile microtubes for saliva handling and storage. Importantly, a critical step in the diagnostic procedure is saliva pretreatment, which addresses the complex viscoelastic nature of saliva.^[^ ^66^, ^67^, ^68^ ^]^ This process involves the removal of impurities to minimize nonspecific adsorption on the electrode surface, while simultaneously preserving target biomarkers (i.e., MMP‐8) for accurate and reliable detection, as it directly impacts the sensitivity and specificity of the diagnostic assay. Figure 4c illustrates the details of the biosensing workflow for the impedance‐based MMP‐8 detection mechanism, including saliva collection. A super‐absorbent pad (Super SAL, Oasis Diagnostics, Vancouver, WA, USA) collected >1 mL saliva within 1–3 min (indicated by a color change from yellow to blue) and a centrifugation‐dependent system (i.e., Salivette, Sarstedt, Nümbrecht, Germany), as shown in Figure 4d and Figure S19 (Supporting Information), demonstrating their effectiveness in retaining MMP‐8 biomarkers for subsequent biosensing.

Figure 4e presents the normalized impedance responses (Δ*R*_ct_/*R*_ct (blank)_) by applying the standard MMP‐8 sample in a wide range of concentrations (1–600 ng mL⁻¹), where EIS confirmed a linear sensitivity (0.1 V potential, 10 mV amplitude, 100 kHz–0.05 Hz range), consistent with earlier studies. To confirm the reliability, differential pulse voltammetry (DPV) was also employed as a complementary method, demonstrating a concentration‐dependent linear response in redox probe signals (Figure S20, Supporting Information). The MIP‐based electrode exhibited a strong correlation (*R* ^2^ = 0.8669) between Δ*R*_ct_ and MMP‐8 concentration, significantly outperforming the non‐imprinted control (*R* ^2^ = 0.6381), as summarized in Figure 4f. These results collectively validate the sensor's precision in artificial saliva, corroborating the critical role of MMP‐8 imprinted cavities in selective recognition and its applicability in clinical diagnostics. Next, to further assess the analytical performance of the sensor under clinically relevant conditions, pretreated saliva from healthy individuals was spiked with MMP‐8 at concentrations of 0, 100, and 200 ng mL⁻¹. The sensor exhibited a linear concentration‐dependent increase in Δ*R*_ct_, with a stable and low response at 0 ng mL⁻¹, confirming minimal nonspecific interactions (Figure 4g). The observed response patterns in spiked saliva closely match those in artificial saliva, further confirming the biosensing reliability and consistency across different matrices. Following this validation, clinical testing was conducted using saliva samples from three distinct cohorts (healthy individuals, periodontitis patients, and recovered individuals, *n* = 20 per cohort). MMP‐8 concentrations in all samples were quantified using an ELISA kit (Figure 4h; Figure S21, Tables S4–S10, Supporting Information). As previously reported, salivary MMP‐8 concentrations exceeding 200 ng mL⁻¹ serve as a diagnostic threshold for periodontitis in Korean populations, accounting for ethnic and geographical variability.^[^ ^2^, ^69^, ^70^, ^71^ ^]^ In Figure 4i, a scatter plot presents Δ*R*_ct_/*R*_ct (blank)_ values for MMP‐8 detection in MIP and NIP electrodes across patient samples, where the MIP electrodes exhibited significantly higher responses compared to NIP, confirming the selective molecular recognition capability of the imprinting process (*p* < 0.05). Indeed, the sensor demonstrated comparable sensitivity in patient‐derived samples (≈200 ng mL⁻¹) to standardized MMP‐8 solutions, validating its clinical applicability. Figure 4j validates the clinical utility of the sensor through Parkes error grid analysis,^[^ ^72^ ^]^ correlating its performance with ELISA across standardized calibrants (red data points) and clinical saliva samples (gray data points). The MIP sensor responses to standardized MMP‐8 solutions (50–400 ng mL⁻¹, red) demonstrated high analytical precision, with >95% of measurements clustered in clinically reliable Zones A/B (deviations <20% from reference values). Concurrently, the sensor readings from ELISA‐prevalidated clinical samples (gray) exhibited strong concordance (*r* = 0.92). This dual‐validation approach, combining controlled calibrant testing (red) and clinical correlation (gray), confirms the sensor's accuracy across diverse matrices, with no data observed in error‐prone Zones D/E. These findings highlight the fidelity of the MIP‐based sensing platform as a diagnostic tool for periodontitis, establishing its potential as a complementary technology for periodontitis monitoring.

Figure 4k presents a structural design of the wearable mouthguard‐integrated diagnostic device, comprising multiple key components optimized for real‐time salivary biomarker monitoring. An array of functional chip modules, including a wireless chip, a compact data acquisition and transformation unit, and a magnet‐assisted guide plate, was fully integrated onto a flexible printed circuit board (FPCB) to facilitate robust electrical interfacing between the MIP electrodes and the signal acquisition system. Comprehensive details regarding the circuit layout, surface‐mounted component integration, and the fabrication of the prototype POCT platform are provided in Figures S22–S24 (Supporting Information and Experimental Section). For stable biomarker collection, a biocompatible silicone‐based mouthpiece incorporates a via hole that channels saliva through a microfluidic transport pathway toward the sensing interface (i.e., the MIP electrode). The filter membrane supported by an O‐ring assembly, enhances sample stabilization and contaminant filtration, thereby aiding high‐fidelity biomarker detection. Figure 4l presents a cross‐sectional schematic illustrating the intraoral placement and fluidic dynamics of the mouthguard device. The MIP electrode, embedded within the mouthguard, is interfaced with a porous hydrogel membrane, which facilitates saliva absorption and controlled biomarker diffusion. To ensure its safe application in intraoral environments, the cytocompatibility of the MIP‐modified electrode was evaluated using L929 fibroblast cells, as detailed in the Experimental Section. As shown in Figure 4m, the hydrogel network serves as a biochemical pre‐filtration unit, modulating MMP‐8 transport dynamics while preventing non‐specific adsorption.^[^ ^73^, ^74^ ^]^ The via hole architecture, integrated into the mouthpiece, allows a continuous and uninterrupted biomarker collection process, optimizing real‐time diagnostic feasibility.

A key feature of the pretreatment system is the equipment of the hydrogel‐based filtration unit, which facilitates MMP‐8 diffusion while excluding interfering other contaminants (Figure 4n). The porous polyvinyl alcohol (PVA) hydrogel matrix provides a highly selective molecular sieve, enhancing efficient biomarker interaction with the MIP electrode. Importantly, the hydrophilic properties of the hydrogel promote rapid saliva absorption, ensuring stable biomolecule retention and diffusion, thereby improving sensor performance and analyte recognition fidelity. Figures 4o and S25 (Supporting Information) present SEM images of the hydrogel membrane with a highly interconnected porous structure. As observed in the magnified inset image, the uniform pore size distribution (≈2–5 µm) and a controlled spatial network facilitate efficient biofluid diffusion while maintaining membrane integrity. The membrane thickness of ≈700 µm ensures reproducible and consistent sample processing, effectively eliminating solid particulates and cellular debris while preserving analyte transport efficiency toward the MIP electrode interface. To optimize the hydrogel's swelling behavior, Figure 4p presents swelling kinetics for PVA‐based membranes incorporating varying concentrations of potassium hydroxide (KOH) and sucrose. The swelling ratio, ((*W*_s_ − *W*_d_)/*W*_d_ × 100), where *W*_s_​ and *W*_d_​ denote swollen and dried membrane weights), was continuously monitored over time. The results indicate a rapid absorption phase within the first ≈50 min, followed by a stabilization plateau, confirming an optimal balance between hydrogel hydration and mechanical durability. Figure 4q displays EIS data, representing concentration‐dependent responses of normalized Δ*R*_ct_/*R*_ct (blank)_ values in both PBS and spiked saliva samples, through the hydrogel membrane. The wearable sensing platform demonstrated significantly higher sensitivity compared to NIP controls, confirming the high selectivity of the MMP‐8 imprinted cavities. Among the tested formulations, PVA/KOH/10% sucrose (swelling ratio ≈600%) exhibited a stable sensing response, demonstrating an optimized balance of hydrophilicity and biomolecule retention efficiency. The correlation between membrane swelling capacity and structural integrity affirms that the porous hydrogel matrix remains intact during hydration, supporting stable MMP‐8 diffusion. The hydrogel membrane's protein recovery rate was validated at ≈80%, as confirmed by depressurized filtration experiments (Figure S26, Supporting Information). This high recovery efficiency ensures selective rebinding of MMP‐8 within the MIP‐imprinted cavities, sufficiently guiding biosensor sensitivity and reproducibility. Furthermore, the detached MIP electrode was systematically assessed for reusability, wherein fresh electrolyte solutions were applied following each measurement cycle. Electrochemical characterization revealed a 60% and 50% increase in Δ*R*_ct_/*R*_ct (blank)_ at 600 ng mL⁻¹ in PBS and saliva matrices, respectively. These impedance variations were substantially greater than the ≤20% fluctuations observed in NIP controls, further validating the sensor's specificity and diagnostic accuracy. This set of experiments demonstrates the wearable integration of MIP biosensor supported by a hydrogel‐based salivary sampling system, emphasizing its structural robustness, efficient sample processing. The wearable format and selective biomarker detection capabilities collectively establish this platform as a promising solution for non‐invasive, POCT of periodontal inflammation, offering an adaptable framework for precision diagnostics and personalized healthcare applications.

### 2.5 Implementation and Integration of Clinical Data‐Driven DL Models for Periodontitis Diagnosis

**Figure 5:**
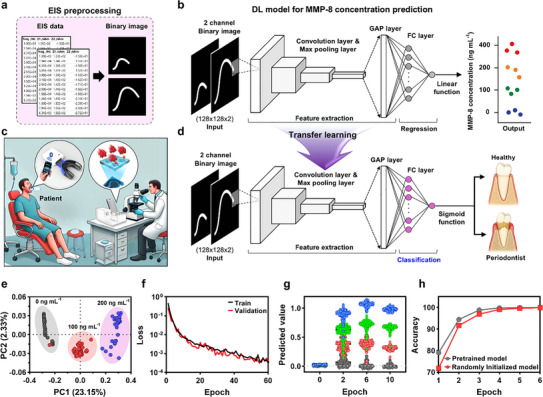
DL‐driven EIS data processing. a) Preprocessing pipeline for EIS data, converting raw spectra into normalized two‐channel images. b) Architecture of the CNN regression model for MMP‐8 concentration prediction. c) Conceptual schematic of the POCT platform integrated with an intraoral diagnostic device for on‐site clinical evaluation. d) TL framework for clinical classification utilizing pretrained models. e) PCA plots visualizing the separability of different MMP‐8 concentrations in DL‐based classification of standardized EIS data. f) Training curves depicting the learning progression of the regression model. g) Predicted values of the preclinical DL model across different MMP‐8 concentrations over training epochs. h) Comparison of training performance between a pre‐trained model and a randomly initialized model.

EIS data obtained from periodontal disease patients (Figure 4i) were utilized as input parameters for the advanced DL model developed in this study, facilitating precise prediction of periodontitis severity. Specifically, during clinical assessments, the concentrations of the MMP‐8 biomarker were quantitatively determined, establishing an effective method for predicting periodontitis risk. This biomarker‐centric diagnostic strategy significantly elevates the accuracy and reliability of periodontitis assessments via the biosensor platform presented herein. **Figure** **5**a outlines the preprocessing pipeline employed on the raw numerical EIS datasets, transforming them into two‐channel binary image representations (Figure s27, Supporting Information). This transformation produces structured arrays with a shape of (128, 128, 2), substantially enhancing the capability of the DL model to efficiently identify and extract critical features from impedance spectra.^[^ ^75^ ^]^ The binary images generated preserve vital impedance variations across different frequency domains, thus enabling the DL model to accurately discern biomarker‐specific electrochemical signatures. Figure 5b illustrates the specifically tailored DL architecture designed to regress MMP‐8 biomarker concentrations accurately. Employing a convolutional neural network (CNN)‐based framework, this model directly processes two‐channel binary EIS images, capitalizing on convolutional and max‐pooling layers for optimal feature extraction. The initial stage of the DL architecture comprises three to five convolutional layers coupled with rectified linear unit (ReLU) activation functions and pooling layers, effectively extracting informative feature maps. These extracted features subsequently undergo refinement through a global average pooling (GAP) layer and are further processed by fully connected (FC) layers. Ultimately, linear output layers provide precise predictions of periodontitis risk, quantitatively represented by MMP‐8 concentrations. A sliding‐window‐based approach was employed to systematically handle sequential data structures and perform necessary frequency‐range adjustments. Prior to conversion into binary images, the raw EIS data underwent rigorous Min–Max scaling using predefined parameters to normalize the values consistently within a range of 0–1. The scaled numerical data were subsequently converted into two‐channel binary grayscale images using the Python Imaging Library (PIL). This two‐channel image structure explicitly incorporates charge transfer resistance variations, embedding critical initial electrode‐state information directly into the input layers of the DL model. Before incorporating clinical patient data, a robust and comprehensive training dataset was assembled, consisting of four distinct MMP‐8 concentration categories. Consequently, the resulting DL architecture was calibrated to accurately perform regression over a clinically relevant concentration range (0–400 ng mL⁻¹), enabling continuous, real‐time monitoring of periodontal inflammatory states.

Figure 5c presents a conceptual framework illustrating the integration and deployment workflow of a clinically oriented DL model, explicitly designed for seamless incorporation within POCT systems and mouthguard‐based diagnostic technologies. Building on this foundation, Figure 5d elucidates the sophisticated application of transfer learning (TL) methodologies. Here, pretrained DL models, originally developed using extensive standardized analyte datasets, are integrated into clinical diagnostic settings. This strategic use of transfer learning effectively mitigates the prevalent challenge of insufficient clinical data, thus facilitating the development of robust, reliable, and clinically applicable diagnostic tools.^[^ ^76^ ^]^ In this approach, weights from the feature extraction layers of the pretrained DL model, initially trained for MMP‐8 concentration prediction, were systematically transferred to the clinical diagnostic model. Subsequently, a fine‐tuning process was undertaken using a carefully curated and constrained clinical dataset. For the fine‐tuning tailored specifically for classification tasks, all pretrained layers, apart from the final layers dedicated exclusively to regression outputs, were preserved. A dense layer incorporating a sigmoid activation function was then introduced, enabling accurate binary classification (0: healthy, 1: periodontitis). This targeted approach strategically capitalizes on the pretrained model's existing knowledge base, significantly enhancing diagnostic precision even in contexts characterized by limited clinical data availability.

Figure 5e presents a principal component analysis (PCA) plot, systematically comparing the performance of the DL‐based methodology against conventional machine learning (ML) models (Figure S28, Supporting Information). Notably, the DL approach effectively captured intricate patterns inherent in EIS data, exhibiting significantly enhanced classification performance. This facilitated rigorous statistical validation and direct comparative analyses using identical datasets. To robustly evaluate the efficacy of the DL model, comparisons were drawn with traditional ML techniques based on dielectric relaxation theory, specifically employing an ECM. The ECM methodologically simplifies complex electrode–electrolyte interface dynamics into fundamental electrical components (Figure S29, Note S1, and Table S11, Supporting Information). Conventional ML models traditionally employ manually selected feature values derived directly from EIS data.^[^ ^77^, ^78^ ^]^ Although widely utilized in applications such as secondary battery diagnostics, these classical methods inherently suffer limitations due to insufficient capture of nonlinear relationships within complex electrochemical datasets, resulting in notable overlaps among data clusters in PCA plots. This observation demonstrates classical ML models’ inability to adequately reflect the multidimensional complexity intrinsic to electrochemical data. Conversely, the developed DL approach innovatively transforms EIS datasets into two‐channel binary image inputs, enabling automatic extraction of electrochemical features via CNNs. By this, our DL model proficiently captured nonlinear data correlations, effectively representing high‐dimensional electrochemical features.^[^ ^79^ ^]^ Consequently, PCA analysis distinctly demonstrated improved separation in a given analyte concentration range, clearly delineating classification boundaries and highlighting the DL model's superior capacity to capture intricate data patterns. In quantitative detail, as summarized in the 2 × 2 canonical cross‐tabulation (Table S12, Supporting Information), traditional ML approaches achieved reasonable accuracy (≈89.6%) only in distinguishing between widely separated biomarker concentrations (0 vs 200 ng mL⁻¹ MMP‐8). However, their performance significantly deteriorated when distinguishing intermediate concentrations (i.e., 0 vs 100 ng mL⁻¹ (≈71.8%) and 100 versus 200 ng mL⁻¹ (≈68.9%)), highlighting intrinsic limitations in capturing nuanced electrochemical characteristics due to reliance on manually selected, low‐dimensional features. These shortcomings were visually evident through considerable overlaps within PCA cluster distributions (Figure S30, Supporting Information).^[^ ^80^ ^]^ The classification performance of the DL model was evaluated using confusion matrices across the same MMP‐8 concentration pairs (0 vs 100, 0 vs 200, and 100 vs 200 ng mL⁻¹), as summarized in Table S13 (Supporting Information). Notably, the DL model exhibited outstanding accuracy, surpassing ≈99.5% across all assessed concentration range. The DL‐driven PCA analyses notably demonstrated superior cluster separability, reinforcing clear delineation of classification boundaries and significantly outperforming classical ML methods. Thus, the DL model's capability to autonomously extract and interpret complex nonlinear electrochemical signatures provides a substantial technical advancement, representing a critical enhancement in disease prediction and diagnostic accuracy utilizing EIS data.

The connectivity and predictive effectiveness of the DL‐based model were thoroughly evaluated through an extensive analysis of training dynamics and diagnostic accuracy. Specifically, Figure 5f presents the loss of the regression model across training epochs, exhibiting a stable learning curve indicative of robust training without evidence of overfitting. Remarkably, predictive accuracy consistently surpassed ≈99% after merely 10 epochs, demonstrating the model's capability for rapid convergence and stable learning, as further supported by epoch‐wise monitoring depicted in Figure 5g. Accuracy metrics were meticulously derived by discretizing both the predicted regression outputs and the corresponding ground‐truth concentrations into clearly defined categorical labels of {0, 1/3, 2/3, and 1}. This discretization strategy facilitated precise, quantitative evaluations of diagnostic performance throughout the training epochs. Additionally, Figure 5h emphasizes the substantial benefit conferred by employing TL for clinical dataset integration. Utilizing a pretrained DL model initially trained on extensive laboratory‐derived standard samples, TL achieved exceptional accuracy exceeding ≈99% within the initial epoch. This outcome effectively mitigates typical challenges posed by limited clinical datasets, substantially enhancing model reliability. While both conventional training and TL methodologies attained ≈100% accuracy by the third epoch, the TL‐based approach distinctly accelerated the model's convergence process. This feature highlights the superior suitability of transfer learning for practical deployment in resource‐constrained computational environments.^[^ ^81^ ^]^

Thus far, we have demonstrated the advantages of DL methodologies in deciphering complex electrochemical datasets, thereby reinforcing their potential as robust diagnostic frameworks in biomedical sensing applications. Nevertheless, it is essential to acknowledge that, due to the relatively limited dataset size, further validation using extensive and diverse clinical cohorts is imperative to robustly establish the model's generalizability and clinical applicability. Therefore, future work should focus on comprehensive dataset expansion to mitigate overfitting and enhance model generalizability by addressing challenges posed by data acquisition biases, batch effects, and image variability (e.g., contrast variations).^[^ ^82^ ^]^ Furthermore, as reliance solely on a single data modality, such as EIS, may limit model robustness, integrating multimodal data sources is recommended. Developing hybrid models that incorporate complementary analytical modalities, including imaging, biochemical assays, and clinical metadata, could significantly enhance diagnostic accuracy and reliability, particularly for complex multifactorial diseases such as periodontitis (Figure S31, Supporting Information).^[^ ^83^, ^84^ ^]^ Additionally, as highlighted, addressing non‐biological confounders and biases in data acquisition through advanced data augmentation techniques and statistical methodologies will be crucial for translating DL‐driven diagnostic models into clinical practice.

## 3 Conclusion

In summary, we engineered an advanced electrochemical sensing platform by integrating a poly(o‐PD)‐based molecularly imprinted recognition layer onto a GO‐modified electrode surface, achieving selective and non‐invasive detection of MMP‐8 in saliva while simultaneously enhancing molecular recognition specificity and electrochemical sensitivity. Through a combination of DFT simulations and experimental validations, we demonstrated that the molecular imprinting design provided targeted binding affinity and high selectivity toward MMP‐8 over closely related interferents. The integration of a GO interlayer beneath the MIP electrode was crucial for enhancing sensor uniformity and signal stability, enabling the reproducible fabrication of high‐performance molecularly imprinted electrodes. Furthermore, the MIP‐functionalized electrodes were incorporated into a wearable mouthguard platform, equipped with a pre‐filtration system, facilitating real‐time biomarker monitoring without the need for external preprocessing. Complementary DL‐driven analysis frameworks further improved classification robustness, thereby supporting clinical applicability. Collectively, this study establishes an integrated strategy combining computational design, nanomaterial engineering, and wearable system development to advance practical molecular diagnostics in oral healthcare. We believe that this approach highlights the strength of integrating MIP‐based molecular capture, nanomaterial‐enhanced signal transduction, wearable device implementation, and artificial intelligence analytics, offering a promising route toward personalized oral health monitoring.^[^ ^85^, ^86^, ^87^ ^]^

## 4 Experimental Section

### Materials

o‐Phenylenediamine (o‐PD, 98%), sulfuric acid (H₂SO₄, 98%), methanol (99%), potassium hexacyanoferrate (K₃[Fe(CN)₆]), potassium hexacyanoferrate trihydrate (K₄[Fe(CN)₆]⋅3H₂O), phosphate buffered saline (PBS, 0.01 m, pH 7.4), poly(vinyl alcohol) (PVA, 89000‐98000 Mw), potassium chloride solution (KCl, 0.1 m), sucrose (99.5%) and artificial saliva were purchased from Sigma‐Aldrich Co. (St. Louis, MO, USA). Graphene oxide (GO, 6.2 mg mL⁻¹ aqueous dispersion) was purchased from Graphene Supermarket Inc. (Calverton, NY, USA). Matrix metalloproteinase‐8 (MMP‐8) was purchased from Sino Biological Inc. (Beijing, China), Tumor necrosis factor alpha (TNF‐α), Matrix metalloproteinase‐1 (MMP‐1), Matrix metalloproteinase‐9 (MMP‐9), and Interleukin 1 beta (IL‐1β) were purchased from NKMAX (Seongnam, Korea).

### Semi‐Automated MIP‐Functionalized Electrode Fabrication and Process Optimization

The semi‐automated MIP‐based electrode fabrication system was integrated into an XYZ‐axis precision motion stage (DTR Series SU, Hyulim Robot, Cheonan, Korea), with an aluminum frame mounted on the *Z*‐axis, integrating an electrode connector, cleaning module, and drying module. The connector was interfaced with electrochemical instrumentation to facilitate real‐time process control, allowing for precise monitoring and adjustment during fabrication. The fabrication process consisted of three sequential stages: SPCE pretreatment, GO deposition, and o‐PD polymerization with MMP‐8. Following each step, electrodes were autonomously transferred to the cleaning module for residual impurity removal in DI water, followed by nitrogen gas drying. After fabrication, the electrodes were manually detached and subjected to template extraction using a within dedicated flow cell apparatus. Finalized MIP electrode underwent a controlled drying protocol (30 °C, 10‐min) and was stored under a nitrogen gas atmosphere to prevent oxidative degradation. MIP‐functionalized electrodes were synthesized via electropolymerization using a CompactStat potentiostat (Ivium Technologies BV, Eindhoven, Netherlands) under optimized electrochemical parameters. SPCEs were initially activated in 0.5 m H₂SO₄ via CV cycles between −0.6 and 0.8 V at a scan rate of 100 mV s^−1^. Following surface activation, a uniform GO layer was deposited onto the SPCEs through 10 CV cycles (−0.6 to 0.8 V, 100 mV s^−1^) in a 5.2 mg mL^−1^ GO dispersion. To fabricate the MIP‐based sensing layer, GO‐modified SPCEs were immersed in 0.01 m PBS containing 5 mm o‐PD and 0.14 µm MMP‐8 template, followed by electropolymerization via CV over 20‐cycles between 0 and +1.1 V at a scan rate of 50 mV s⁻¹. The template (MMP‐8) was extracted by immersing the electrodes in a 1:1 (v/v) methanol/DI water solution under stirring at 300 rpm for 60‐min. In the case of subsequent reuse cycles, surface‐bound MMP‐8 was selectively detached using a brief 5‐min extracting protocol to preserve the integrity of the imprinted matrix. For the preparation of the NIP electrode, an identical procedure was followed, except that the MMP‐8 template was omitted during the electropolymerization step. GO‐modified SPCEs were immersed in 0.01 m PBS containing only 5 mm o‐PD and subjected to the same CV conditions. After polymerization, the NIP electrodes were treated with the same methanol/water extraction step to mimic the post‐processing conditions of the MIP fabrication. Since no template molecule was present, the resulting NIP film lacks molecular recognition sites, serving as a non‐selective reference for evaluating the MIP‐based sensor's specificity and selectivity.

### Electrochemical Characterization and Performance

The performance of MIP and NIP electrodes was evaluated in 0.1 m KCl solution, containing 5 mm K₃[Fe(CN)₆]/K₄[Fe(CN)₆] redox probe, using a potentiostat. Three complementary techniques were employed under ambient conditions (25 ± 0.5 °C): CV (−0.6 to +0.8 V, 100 mV s⁻¹), EIS (100 kHz–0.05 Hz, 10 mV amplitude, DC bias = 0.1 V), DPV (0.4–1 V window, 0.01 V step, 0.05 s pulse width). Direct detection capabilities were validated in 0.01 M PBS (pH 7.4), artificial saliva, and clinical saliva. Target adsorption kinetics were quantified by incubating sensors in MMP‐8 solutions (1–1000 ng mL⁻¹ in PBS) for 1 h before EIS analysis. Cross‐reactivity was assessed against MMP‐1, TNF‐α, and MMP‐9 (200 pg mL⁻¹ each), using single and dual protein mixtures. The complete electrochemical EIS measurement protocol for each MIP sensor followed a standardized sequence: i) For baseline measurement, the MIP sensor was first applied to PBS solution to record the baseline impedance (*R*_ct(blank)_). The sensor was then rinsed with DI water and dried. ii) For analyte measurement, the same MIP electrode was incubated statically for 60 min in either the MMP‐8 test solution or pretreated patient saliva. iii) After incubation, the sensor was rinsed again with DI water, dried, and covered with the redox probe, and EIS measurement was performed to record the impedance response for the MMP‐8 sample. Δ*Rct* values were separately measured by exposure to varying analyte concentrations, which could be normalized against this baseline to quantify the sensor's responses.

### MD and DFT Simulations for Structural and Electrostatic Analysis

The structural conformation of the o‐PD/MMP‐8 prepolymerization complex was predicted using MD simulations in GROMACS (University of Groningen, Netherlands). The MMP‐8 structure was modeled using an AlphaFold‐predicted structural model (UniProt: P22894, Google DeepMind, USA), excluding the pre‐domain region (residues 1–108). A total of 2500 o‐PD monomers (122.419 g mol⁻¹) were randomly distributed within a 10 nm^3^ periodic simulation box centered on MMP‐8, achieving 42.21% volumetric occupancy relative to close‐packing density. Simulations employed the CHARMM36 force field (MacKerell Lab) with explicit TIP3P solvation under periodic boundary conditions. Energy minimization (1000 steps, steepest descent) was performed to eliminate steric clashes and stabilize initial atomic configurations. The resulting molecular structures were visualized using SAMSON software (OneAngstrom, France), and hydrogen bonding between o‐PD and MMP‐8 was analyzed using the Hydrogen Bond Finder tool. DFT simulations (B3LYP/6‐31+G, Gaussian 16W, Gaussian, USA) computed ESP distributions, which were mapped onto van der Waals surfaces using GaussView 6.0.1 (Gaussian, USA). Binding energies were also determined by integrating ESP distributions over protein–ligand interfaces using a surface integral approach.

### Fabrication and Characterization of Flexible Sensor Platforms

Flexible substrates were fabricated on a polyimide (PI) sheet (initial dimensions: 55 × 42 mm, thickness: 0.25 mm) sputter‐coated with 12 µm Cu layers on both surfaces. Electrical interconnections were established via primary and secondary metallization, which incorporated precision via‐hole patterning. An SMT stencil was precisely positioned on the prepared FPCB substrate, and solder paste was selectively applied to contact pads via stencil printing with a squeegee blade (Figure S22, Supporting Information). Electronic components were then surface‐mounted on the FPCB and reflow‐soldered at 250 °C for 10‐min under a controlled thermal profile. Interconnect stability and electrical performance were evaluated using a semiconductor parameter analyzer (Keithley 4200A, Beaverton, OR, USA), while mechanical durability was assessed via cyclic bending tests under controlled strain conditions. The device integrated a commercial potentiostat chip, a wireless communication module, and a 5 V Li‐ion battery, with USB and power connectors. The external smartphone was connected via bluetooth low energy for real‐time data transmission. POCT platform housings and mouthguard‐integrated diagnostic device were fabricated using an FDM 3D printer (M160, Moment, Seoul, Korea) with polylactic acid (PLA) filament with a 100 µm layer thickness. High‐resolution components were fabricated via stereolithography (Form3, Formlabs, Massachusetts, USA) using biocompatible photopolymer resins for enhanced structural fidelity.

### Development of a Mobile Application for Wireless Data Processing and Acquisition

The Android application was implemented in Android Studio, integrating open‐source electrochemical sensor chip libraries optimized for compatibility with the POCT platform. The application established Bluetooth‐based wireless communication with the POCT platform and wearable devices, supporting command execution, biomarker data acquisition, signal processing, and data visualization.

### Clinical Validation: Human Subject Recruitment and Sample Processing

MMP‐8 biosensor validation was conducted under an Institutional Review Board (IRB) approved protocol (PNUDH 2022‐10‐009) from Yangsan Hospital, Pusan National University, in compliance with ethical and regulatory guidelines. Eligible participants (≥18‐year‐old) were recruited via advertisements at the Campus and in surrounding communities. All participants provided written informed consent before saliva collection. Samples were immediately filtered, aliquoted into cryotubes, and stored at −80 °C for subsequent analysis. MMP‐8 concentrations in human saliva were quantified using a human MMP‐8 ELISA Kit (ab219050, Abcam, Cambridge, MA, USA) following the manufacturer's protocol, with a detection limit of 19.5 pg mL^−1^.

### Data Acquisition, Preprocessing, and Augmentation

To train the preclinical DL model and the diagnostic DL model, the values of the real and imaginary parts of the impedance, except for the frequency information in the EIS data, were used. The input data (i.e., 2‐channel binary image) was prepared by EIS measurement before and after binding specific analytes, and the output data was prepared as standard MMP‐8 concentration or patient disease presence to be used as training data. The EIS data was preprocessed into binary image data using the PIL and min‐max scaling. The frequency range of the EIS data was adjusted using a similar approach to the sliding window method to further increase the amount of the dataset. Specifically, the frequency range was set from 100 kHz up to 3 Hz by default, and the data were augmented by changing the maximum frequency range. As a result, 7600 sets of data were prepared for the preclinical DL model and 3800 sets for the diagnostic DL model, and each dataset was divided into training (80%) and validation (20%) sets.

### DL Architecture and Performance

The architecture of the DL model was primarily built by combining the CNN layer, the dense layer, and the GAP 2D layers. Additionally, dropout layers were used in the model to prevent overfitting. The preclinical DL and diagnostic DL models used the ReLU as the activation function and employed linear regression and sigmoid function, respectively. The loss function for the diagnostic DL model was binary cross‐entropy, while for the preclinical DL model, it was set to mean squared error. Both models use the Adam optimizer with a learning rate of 10^−3^. The DL models were trained and fine‐tuned using the TensorFlow/Keras framework in a Google Colab environment.

### Cell Culture and Cytocompatibility Assessment

To evaluate the biosafety of the fabricated MIP electrodes for intraoral application, an in vitro cytotoxicity assay was conducted using L929 fibroblast cells. Prior to cell seeding, Both MIP and NIP electrodes were prepared. In the following, fibronectin was coated on the electrodes to promote cell adhesion. Subsequently, L929 cells (3 × 10^3^ cells per well) were seeded onto 96‐well plates (control group) and the surface of the MIP electrodes, then incubated for 48 h under standard culture conditions. Following incubation, the culture medium was removed, and a CCK‐8 solution (10% of the total volume) was added to each well and incubated for an additional 2 h at 37 °C. Cell viability was quantified by measuring optical density (OD) at 450 nm using a microplate reader (SpectraMax 340, Molecular Devices, Sunnyvale, CA). As shown in Figure S32, Supporting Information, the MIP‐modified electrodes maintained ≈80–90% cell viability compared to the control group.

### Statistical Analysis

All data were collected from at least three independent replicates (*n *≥ 3) and were presented as mean ± standard deviation (SD). No additional data transformations, normalizations, or outlier removals were performed. For comparisons between two independent groups, a two‐tailed unpaired *t*‐test was used. Statistical analyses were conducted using GraphPad Prism. Statistical significance is indicated as follows: *p* < 0.05 (^*^), *p* < 0.001 (^***^).

## Conflict of Interest

The authors declare no conflict of interest.

## Supporting information



Supporting Information

Supplemental Movie 1

Supplemental Movie 2

Supplemental Movie 3

## Data Availability

The data that support the findings of this study are available from the corresponding author upon reasonable request;
