# Supplementary material for: A Wearable Electrochemical Biosensor for Salivary Detection of Periodontal Inflammation Biomarkers: Molecularly Imprinted Polymer Sensor with Deep Learning Integration
Source: Adv Sci (Weinh). 2025 Jul 26;12(40):e09658. doi: 10.1002/advs.202509658 (PMC12561418; doi:10.1002/advs.202509658)
Supplement: Supplementary file 1 — Supporting Information [file ADVS-12-e09658-s001.docx]

*Supporting Information*

**A Wearable Electrochemical Biosensor for Salivary Detection of Periodontal Inflammation Biomarkers: Molecularly Imprinted Polymer Sensor with Deep Learning Integration**

*Sangheon Jeon, Sung Hyun Kim, Gyeonghwa Heo, Hye Jin Heo, Seon Yeong Chae, Young Woo Kwon, Shin-Kyu Lee, Dong-Wook Han, Hyun-Joo Kim, Yun Hak Kim* and Suck Won Hong**

S. Jeon, G. Heo, Prof. D.-W. Han, Prof. S. W. Hong.

Department of Optics and Mechatronics Engineering

Department of Cogno-Mechatronics Engineering

College of Nanoscience and Nanotechnology

Pusan National University

Busan 46241, Republic of Korea

E-mail: swhong@pusan.ac.kr (S.W.H.)

S. H. Kim, Y. W. Kwon, S. Y. Chae

Engineering Research Center for Color-Modulated Extra-Sensory Perception Technology

Pusan National University

Busan 46241, Republic of Korea

H. J. Heo,

Department of Anatomy, School of Medicine

Pusan National University

Yangsan, 50612, Republic of Korea

Prof. Y. H. Kim

Medical Research Institute, School of Medicine

Department of Biomedical Informatics, School of Medicine

Periodontal Disease Signaling Network Research Center and Dental and Life Science Institute School of Dentistry

Pusan National University

Yangsan, 50612, Republic of Korea

E-mail: yunhak10510@pusan.ac.kr (Y.H.K.)

S.-K. Lee, Prof. H.-J. Kim

Department of Periodontology, School of Dentistry

Department of Periodontics and Dental Research Institute

Pusan National University Dental Hospital

Yangsan, 50621, Republic of Korea

Keywords: molecularly imprinted polymers, electrochemical biosensor, matrix metalloproteinase-8, point-of-care testing, wearable oral healthcare monitoring device


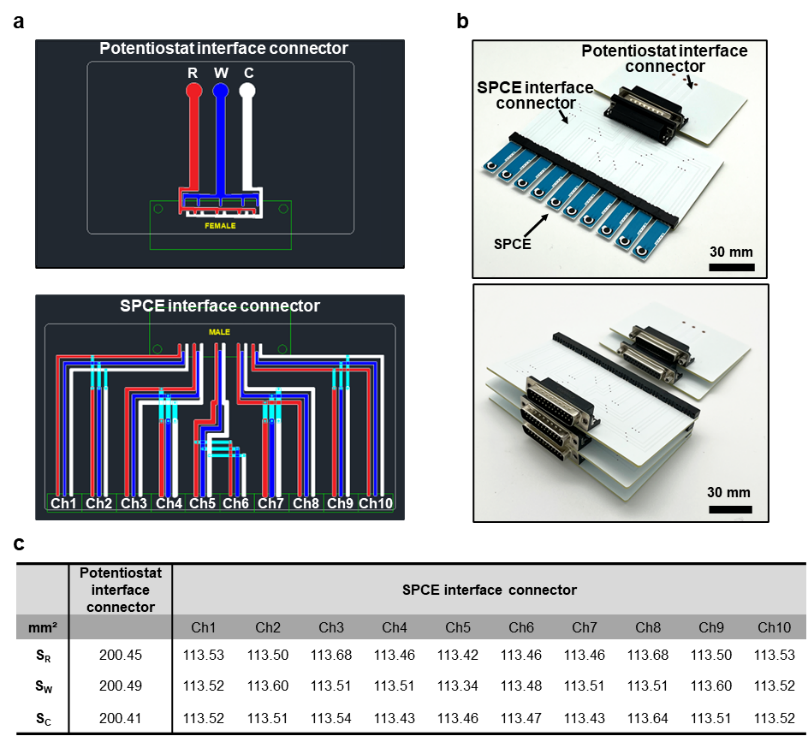


**Figure S1.** a) CAD-rendered design of the upper and lower surfaces of the multi-connector and the corresponding printed circuit board (PCB) layout; the electrical pathway architecture was strategically optimized to ensure uniform current distribution across 10-SPCEs. b) Digital photographs of the fabricated multi-connector modules, demonstrating high-throughput manufacturing process. c) Surface area calculations derived from the CAD-rendered layout in a) with dimensional uniformity across working (W), reference (R), and counter (C) electrodes of the 10-channel SPCE interface.


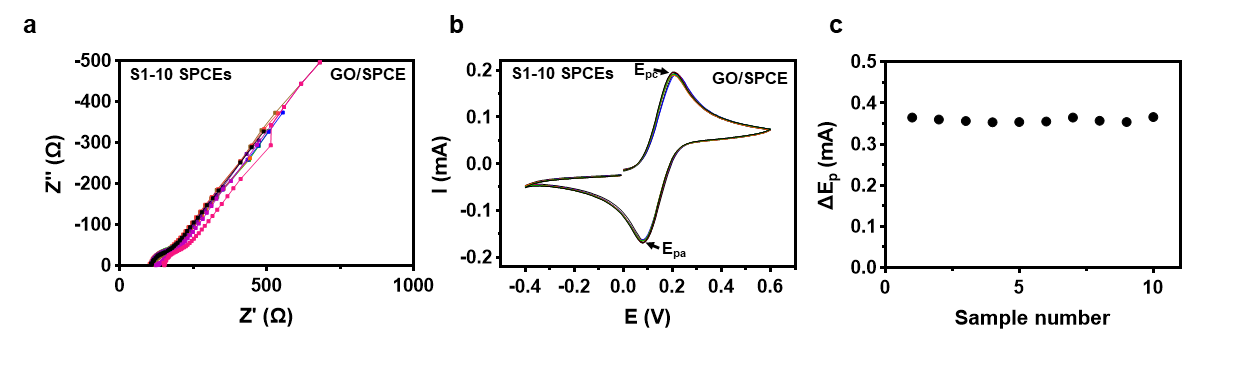


**Figure S2.** Validation of electrode-to-electrode electrochemical reproducibility across GO-modified SPCEs fabricated via the semi-automated multi-terminal system. a) EIS Nyquist plots of ten SPCEs after identical GO deposition, exhibiting highly overlapping impedance profiles and confirming uniform surface modification. b) CV curves of the same SPCEs recorded in K₃[Fe(CN)₆]/K₄[Fe(CN)₆] solution, demonstrating consistent redox peak positions and signal intensities across electrodes. c) Quantitative analysis of peak-to-peak potential differences (Δ*E*_p_) extracted from b), further supporting reproducible electrode behavior with minimal variance. These results validate the structural precision, uniform current distribution, and reliable electrochemical processing achieved by the developed semi-automated fabrication system (refer to Figure S1, Supporting Information).


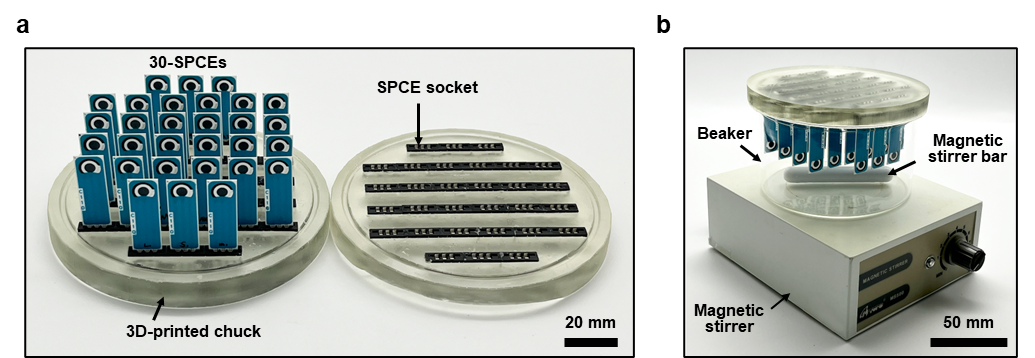


**Figure S3.** Template protein extraction following MIP electrode fabrication. a) Digital photograph of the customized extraction setup designed for the simultaneous removal of template proteins from 30 SPCEs, produced from 3 times semi-automated fabrication system. The system features a 3D-printed chuck fabricated from chemically resistant resin, engineered to vertically secure the SPCEs, ensuring uniform exposure of all electrodes to the extraction solution. b) In a glass beaker, a 1:1 (v/v) mixture of methanol and DI water was placed and stirred at a constant speed using a magnetic stirrer to facilitate consistent and stable template protein extraction.

**Figure S4.** Representative current/voltage profile during the electrochemical process of GO deposition across 10 SPCEs connected in parallel. The curves demonstrate consistent electrochemical behavior among electrodes, confirming uniform GO deposition under identical processing conditions using the semi-automated fabrication system.


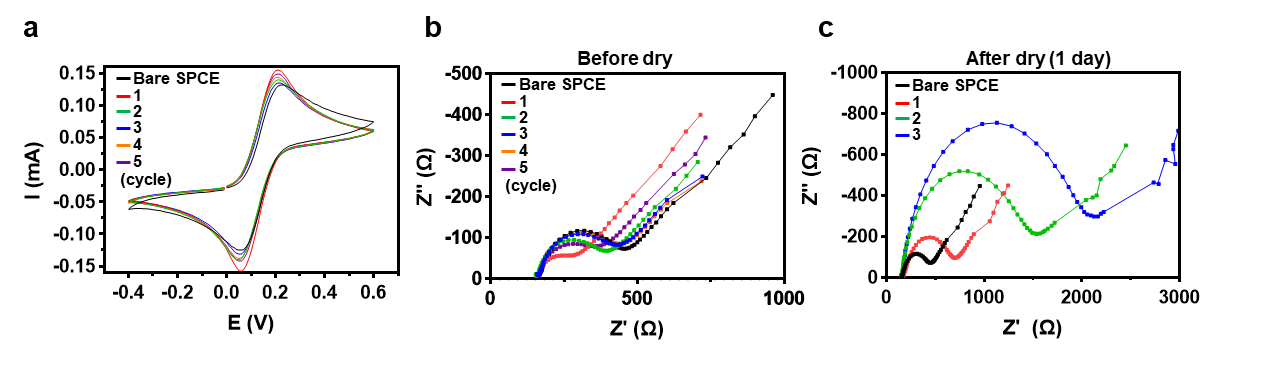


**Figure S5.** Electrochemical characterization as a function of GO deposition cycles. a) CV curves and b EIS Nyquist plots, demonstrating that the overall conductivity slightly increases with successive GO deposition cycles, compared to the bare SPCE. However, beyond a certain number of depositions, the conductivity plateaus, indicating a limit to electrochemical enhancement. c) EIS Nyquist plot obtained after drying the GO film after 1 day, showing an increase in impedance relative to measurements taken immediately after deposition, suggesting a time-dependent change (i.e., complete water molecule removal) in the film’s electrochemical properties.


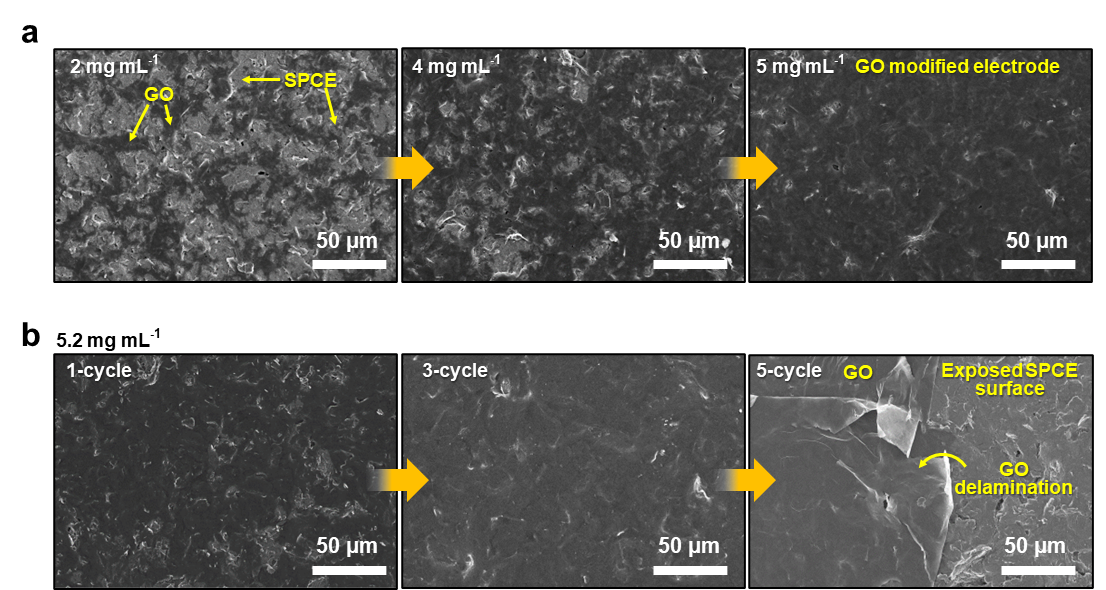


**Figure S6.** Morphological characterization of GO films deposited on the pretreated SPCE surface. a) SEM images depicting the surface morphology of GO films deposited at varying concentrations (i.e., 2, 4, and 5 mg mL⁻¹), highlighting concentration-dependent film formation. b) SEM images illustrating morphological evolution with increasing deposition cycles (i.e., 1, 3, and 5-cycles) at a fixed GO concentration, demonstrating the impact of deposition repetition on film uniformity and surface coverage.


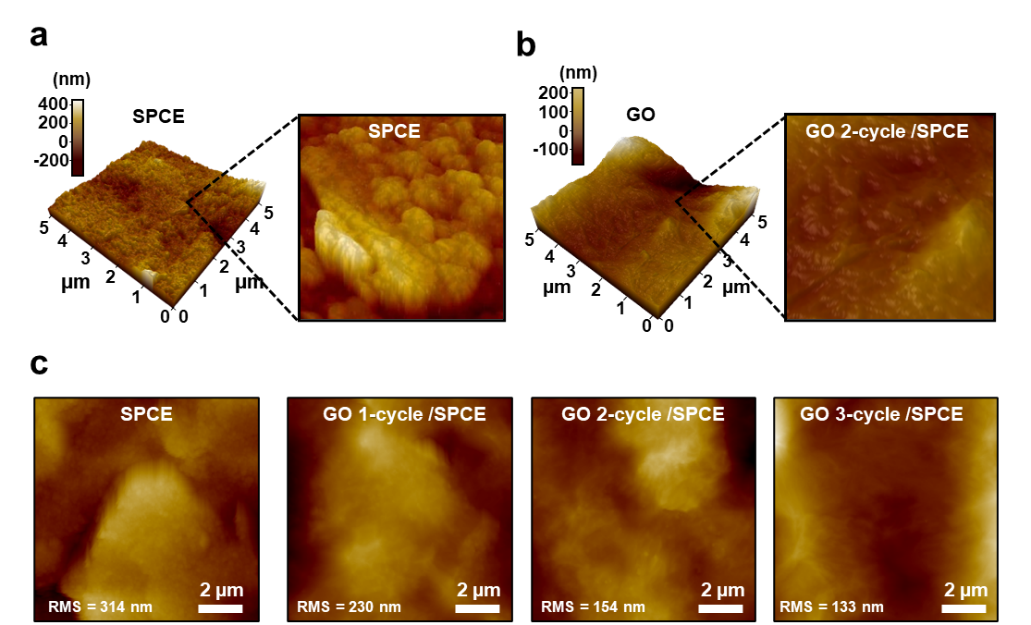


**Figure S7.** AFM characterization of SPCE surface morphology before and after GO deposition. a) Pristine SPCE surface showing relatively high roughness, with RMS value of ≈314 nm. b) SPCE surface after 2-cycle of GO deposition, exhibiting a uniformly coated morphology with stacked GO sheets and a markedly reduced RMS roughness of ≈154 nm. High-resolution AFM images (right) reveal the morphological transition and enhanced surface uniformity following GO modification. c) Quantitative analysis of RMS roughness as a function of GO deposition cycles, indicating a progressive smoothing effect on the SPCE surface. RMS values were obtained from 10 × 10 μm² scan areas.


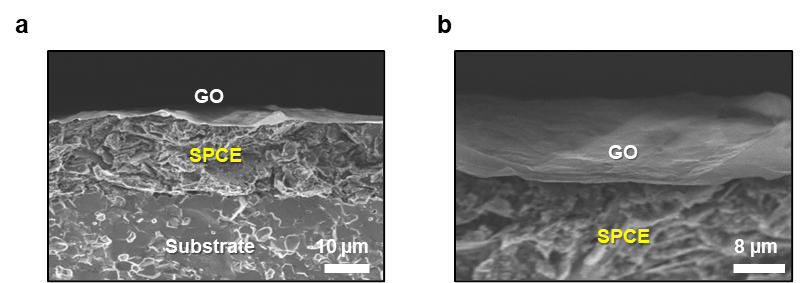


**Figure S8.** Cross-sectional SEM analysis of the GO interlayer, electrochemically deposited on the SPCE surface. a) SEM image revealing the layered structure of the GO interlayer formed on the SPCE surface. b) High-magnification SEM image confirming precise control over the GO interlayer thickness and its homogeneous coverage, facilitating uniform deposition of the subsequent MIP film and contributing to optimized sensor performance.


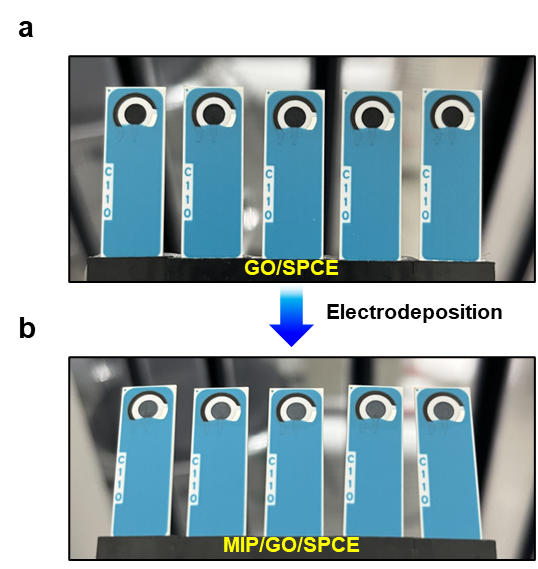


**Figure S9.** Photographic comparison of poly(o-PD)-based MIP electrode. a) GO/SPCEs prior to polymerization. b) MIP/GO/SPCEs, displaying a characteristic gray coloration following polymerization, indicating uniform deposition of the poly(o-PD)-based MIP film on the WE.


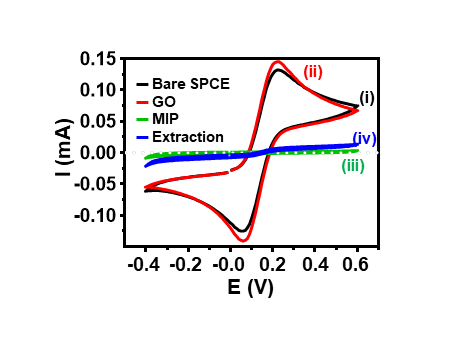


**Figure S10.** Changes of voltammograms during the stepwise-modified SPCE surfaces. CV measurements were performed in 0.1 M KCl containing 5 mM K₃[Fe(CN)₆]/K₄[Fe(CN)₆] at a scan rate of 100 mV s⁻¹ for: i) bare SPCE, ii) GO-modified SPCE (GO/SPCE), iii) MIP-coated GO/SPCE (MIP/GO/SPCE), and iv) after the extraction.

**
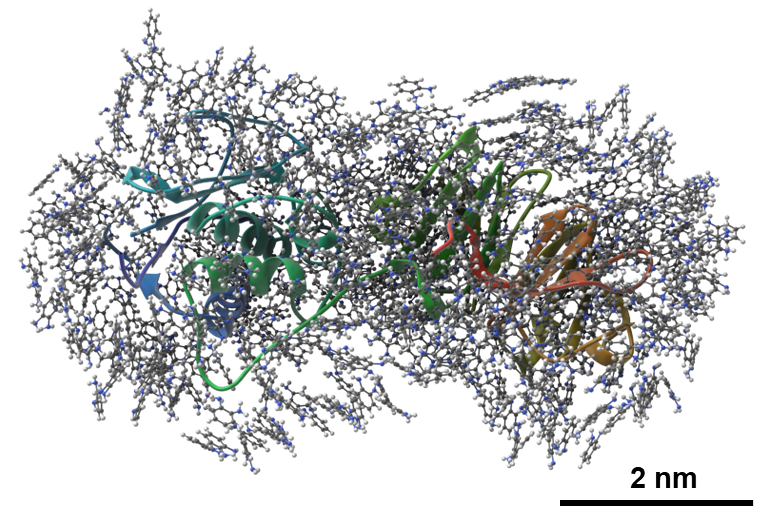
**

**Figure S11**. Schematic illustration of prepolymerizaton complex of o-PD/active-MMP-8 protein calculated by protein-ligand molecular dynamics simulation.

**Table S1**. Hydrogen bond parameters between the o-PD and the 20 amino acid molecules.

| **Molecule**  **structure** | **Energy of the spatial structure  (Hartree)** | **Binding energy of amino acid-EBT**  **(kJ mol^-1^)** | **Hydrogen bond length (Å)** | **Mulliken​ ​charge of targeting oxygen (𝛿)** |
| --- | --- | --- | --- | --- |
| o-PD | -341.113374 | free monomer |  |  |
| Alanine (Ala) | -247.160901 | Template amino acid |  |  |
| 1. o-PD-Ala | -588.239398 | 45.785 | 2.55 | -0.397 |
| Arginine (Arg) | -528.947513 | Template amino acid | - | - |
| 2. o-PD-Arg | -870.046149 | 38.694 | 2.82 | -0.416 |
| Asparagine (Asn) | -414.965116 | Template amino acid |  |  |
| 3. o-PD-Asn | -756.058216 | 53.229 | 2.40 | -0.497 |
| Aspartic Acid (Asp) | -438.18665 | Template amino acid | - | - |
| 4. o-PD-Asp | -779.270959 | 76.311 | 2.50 | -0.461 |
| Cysteine (Cys) | -284.0012695 | Template amino acid |  |  |
| 5. o-PD-Cys | -625.091329 | 61.211 | 1.88 | -0.323 |
| Glutamine (Gln) | -456.345712 | Template amino acid | - | - |
| 6. o-PD-Gln | -797.448248 | 28.455 | 2.58 | -0.289 |
| Glutamic Acid (Glu) | -473.829756 | Template amino acid | - | - |
| 7. o-PD-Glu | -814.934854 | 21.729 | 2.50 | -0.416 |
| Glycine (Gly) | -209.100112 | Template amino acid |  |  |
| 8. o-PD-Gly | -550.191611 | 57.433 | 2.43 | -0.404 |
| Histidine (His) | -470.96581 | Template amino acid | - | - |
| 9. o-PD-His | -812.074946 | 11.126 | 2.65 | -0.407 |
| Isoleucine (Ile) | -365.7579996 | Template amino acid | - | - |
| 10. o-PD-Ile | -706.863256 | 21.312 | 2.61 | -0.357 |
| Leucine (Leu) | -366.306240 | Template amino acid |  |  |
| 11. o-PD-Leu | -707.405291 | 37.604 | 2.55 | -0.314 |
| Lysine (Lys) | -422.096607 | Template amino acid |  |  |
| 12. o-PD-Lys | -763.178176 | 83.503 | 2.21 | -0.323 |
| Methionine (Met) | -725.157334 | Template amino acid | - | - |
| 13. o-PD-Met | -2582.567942 | 53.357 | 2.32 | -0.328 |
| Phenylalanine (Phe) | -479.383724 | Template amino acid | - | - |
| 14. o-PD-Phe | -820.490457 | 17.435 | 2.74 | -0.325 |
| Proline (Pro) | -325.795736 | Template amino acid | - | - |
| 15. o-PD-Pro | -666.908445 | 1.747 | 2.77 | -0.319 |
| Serine (Ser) | -323.584919 | Template amino acid | - | - |
| 16. o-PD-Ser | -664.668674 | 77.765 | 2.33 | -0.273 |
| Threonine (Thr) | -362.886784 | Template amino acid | - | - |
| 17. o-PD-Thr | -703.992602 | 19.838 | 2.58 | -0.365 |
| Tryptophan (Trp) | -569.4559976 | Template amino acid |  |  |
| 18. o-PD-Trp | -910.55702 | 11.565 | 2.44 | -0.401 |
| Tyrosine (Tyr) | -554.556305 | Template amino acid |  |  |
| 19. EBT-Tyr | -895.665775 | 10.250 | 2.69 | -0.303 |
| Valine (Val) | -323.216119 | Template amino acid |  |  |
| 20. o-PD-Val | -664.316592 | 33.871 | 3.11 | -0.264 |

**Table S2**. Hydrogen bond parameters between the EBT and the 20 amino acid molecules.

| **Molecule**  **structure** | **Energy of the spatial structure  (Hartree)** | **Binding energy of amino acid-EBT**  **(kJ mol^-1^)** | **Hydrogen bond Length (Å)** | **Mulliken​ ​charge of targeting Oxygen (𝛿)** |
| --- | --- | --- | --- | --- |
| **EBT** | **-1857.420101** | **free monomer** |  |  |
| Alanine (Ala) | -248.422666 | Template amino acid |  |  |
| EBT-Ala (1) | -2105.837524 | 13.765 | 1.89 | -0.420 |
| EBT-Ala (2) | -2105.833687 | 23.840 | 1.95 | -0.368 |
| EBT-Ala (3) | -2105.813399 | 77.106 | 2.32 | -0.393 |
| EBT-Ala (4) | -2105.830806 | 31.404 | 1.55 | -0.333 |
| **1. EBT-Ala total** |  | **146.115** |  |  |
| Arginine (Arg) | -531.593043 | Template amino acid | - | - |
| EBT-Arg (1) | -2389.006119 | 18.444 | 2.44 | -0.398 |
| EBT-Arg (2) | -2389.004026 | 23.940 | 2.47 | -0.431 |
| EBT-Arg (3) | -2388.980147 | 86.633 | 2.99 | -0.364 |
| EBT-Arg (4) | -2388.984379 | 75.523 | 3.24 | -0.341 |
| **2. EBT-Arg total** |  | **204.54** |  |  |
| Asparagine (Asn) | -417.051539 | Template amino acid |  |  |
| EBT-Asn (1) | -2274.468174 | 9.100 | 2.49 | -0.473 |
| EBT-Asn (2) | -2274.457040 | 38.333 | 2.54 | -0.451 |
| EBT-Asn (3) | -2274.449125 | 59.113 | 2.67 | -0.375 |
| EBT-Asn (4) | -2274.441440 | 79.291 | 2.33 | -0.280 |
| **3. EBT-Asn total** |  | **185.837** |  |  |
| Aspartic Acid (Asp) | -436.346837 | Template amino acid | - | - |
| EBT-Asp (1) | -2293.759472 | 19.602 | 2.03 | -0.277 |
| EBT-Asp (2) | -2293.736226 | 53.798 | 2.33 | -0.316 |
| EBT-Asp (3) | -2293.740248 | 80.635 | 2.62 | -0.384 |
| EBT-Asp (4) | -2293.746447 | 70.075 | 2.80 | -0.285 |
| **4. EBT-Asp total** |  | **224.110** |  |  |
| Cysteine (Cys) | -284.0012695 | Template amino acid |  |  |
| EBT-Cys (1) | -2141.4139045 | 23.333 | 1.94 | -0.410 |
| EBT-Cys (2) | -2141.3906585 | 23.444 | 2.01 | -0.316 |
| EBT-Cys (3) | -2141.3946805 | 77.121 | 2.22 | -0.384 |
| EBT-Cys (4) | -2141.4008795 | 61.211 | 1.88 | -0.323 |
| **5. EBT-Cys total** |  | **185.109** |  |  |
| Glutamine (Gln) | -456.345712 | Template amino acid | - | - |
| EBT-Gln (1) | -2313.763244 | **6.745** | 2.38 | -0.296 |
| EBT-Gln (2) | -2313.734143 | 83.149 | 2.20 | -0.310 |
| EBT-Gln (3) | -2313.722483 | 113.764 | 2.14 | -0.384 |
| EBT-Gln (4) | -2313.754975 | 28.455 | 2.58 | -0.289 |
| **6. EBT-Gln total** |  | **232.113** |  |  |
| Glutamic Acid (Glu) | -475.643492 | Template amino acid | - | - |
| EBT-Glu (1) | -2333.058907 | 12.302 | 2.35 | -0.289 |
| EBT-Glu (2) | -2333.051405 | 32.000 | 2.57 | -0.358 |
| EBT-Glu (3) | -2333.039015 | 64.530 | 2.77 | -0.385 |
| EBT-Glu (4) | -2333.046408 | 45.121 | 2.31 | -0.321 |
| **7. EBT-Glu total** |  | **153.953** |  |  |
| Glycine (Gly) | -209.100112 | Template amino acid |  |  |
| EBT-Gly (1) | -2066.510703 | 24.969 | 2.08 | -0.408 |
| EBT-Gly (2) | -2066.519264 | 2.492 | 2.13 | -0.310 |
| EBT-Gly (3) | -2066.512807 | 19.444 | 2.54 | -0.386 |
| EBT-Gly (4) | -2066.513079 | 18.730 | 2.21 | -0.302 |
| **8. EBT-Gly total** |  | **65.635** |  |  |
| Histidine (His) | -309.9162961 | Template amino acid | - | - |
| EBT-His (1) | -2167.331154 | 25.321 | 2.11 | -0.401 |
| EBT-His (2) | -2167.327317 | 21.851 | 2.31 | -0.358 |
| EBT-His (3) | -2167.307029 | 55.234 | 3.32 | -0.382 |
| EBT-His (4) | -2167.324436 | 44.601 | 4.20 | -0.301 |
| **9. EBT-His total** |  | **147.007** |  |  |
| Isoleucine (Ile) | -365.7579996 | Template amino acid | - | - |
| EBT-Ile (1) | -2223.172858 | 15.3217 | 2.29 | -0.410 |
| EBT-Ile (2) | -2167.327317 | 13.844 | 2.87 | -0.310 |
| EBT-Ile (3) | -2167.307029 | 45.221 | 3.19 | -0.386 |
| EBT-Ile (4) | -2167.324436 | 33.221 | 4.29 | -0.312 |
| **10. EBT-Ile total** |  | **107.607** |  |  |
| Leucine (Leu) | -366.306240 | Template amino acid |  |  |
| EBT-Leu (1) | -2223.718982 | 19.321 | 4.54 | -0.289 |
| EBT-Leu (2) | -2223.721631 | 12.365 | 4.31 | -0.361 |
| EBT-Leu (3) | -2223.725918 | 1.111 | 4.92 | -0.385 |
| EBT-Leu (4) | -2223.726051 | 0.761 | 5.40 | -0.343 |
| **11. EBT-Leu total** |  | **33.558** |  |  |
| Lysine (Lys) | -422.096607 | Template amino acid |  |  |
| EBT-Lys (1) | -2279.493463 | 61.030 | 2.03 | -0.348 |
| EBT-Lys (2) | -2279.507590 | 23.940 | 2.15 | -0.297 |
| EBT-Lys (3) | -2279.491325 | 66.643 | 2.58 | -0.401 |
| EBT-Lys (4) | -2279.510807 | 15.493 | 2.15 | -0.400 |
| **12. EBT-Lys total** |  | **167.106** |  |  |
| Methionine (Met) | -725.157334 | Template amino acid | - | - |
| EBT-Met (1) | -2582.567942 | 24.924 | 2.63 | -0.388 |
| EBT-Met (2) | -2582.564610 | 33.672 | 2.65 | -0.379 |
| EBT-Met (3) | -2582.564858 | 33.021 | 2.65 | -0.388 |
| EBT-Met (4) | -2582.571693 | 15.076 | 2.43 | -0.301 |
| **13. EBT-Met total** |  | **106.693** |  |  |
| Phenylalanine (Phe) | -479.383724 | Template amino acid | - | - |
| EBT-Phe (1) | -2336.794185 | 25.310 | 4.32 | -0.291 |
| EBT-Phe (2) | -2336.803707 | 0.310 | 2.33 | -0.340 |
| EBT-Phe (3) | -2336.803673 | 0.401 | 5.62 | -0.250 |
| EBT-Phe (4) | -2336.803558 | 0.699 | 6.80 | -0.301 |
| **14. EBT-Phe total** |  | **26.72** |  |  |
| Proline (Pro) | -325.795736 | Template amino acid | - | - |
| EBT-Pro (1) | -2183.206075 | 25.630 | 2.25 | -0.393 |
| EBT-Pro (2) | -2183.215328 | 1.337 | 2.84 | -0.281 |
| EBT-Pro (3) | -2183.215603 | 0.615 | 2.22 | -0.320 |
| EBT-Pro (4) | -2183.212880 | 7.764 | 2.39 | -0.311 |
| **15. EBT-Pro total** |  | **35.345** |  |  |
| Serine (Ser) | -323.584919 | Template amino acid | - | - |
| EBT-Ser (1) | -2180.988028 | 44.612 | 2.42 | -0.329 |
| EBT-Ser (2) | -2180.982213 | 59.880 | 2.34 | -0.315 |
| EBT-Ser (3) | -2180.992089 | 33.950 | 2.44 | -0.309 |
| EBT-Ser (4) | -2181.003416 | 4.211 | 2.21 | -0.305 |
| **16. EBT-Ser total** |  | **142.653** |  |  |
| Threonine (Thr) | -362.886784 | Template amino acid | - | - |
| EBT-Thr (1) | -2220.300411 | 16.997 | 2.13 | -0.370 |
| EBT-Thr (2) | -2220.305235 | 4.332 | 2.76 | -0.358 |
| EBT-Thr (3) | -2220.294442 | 32.669 | 2.81 | -0.321 |
| EBT-Thr (4) | -2220.306882 | 0.008 | 2.66 | -0.297 |
| **17. EBT-Thr total** |  | **54.006** |  |  |
| Tryptophan (Trp) | -569.4559976 | Template amino acid |  |  |
| EBT-Trp (1) | -2426.870856 | 11.565 | 2.51 | -0.315 |
| EBT-Trp (2) | -2426.867019 | 25.976 | 2.25 | -0.330 |
| EBT-Trp (3) | -2426.846731 | 67.235 | 2.55 | -0.318 |
| EBT-Trp (4) | -2426.864138 | 42.555 | 2.00 | -0.400 |
| **18. EBT-Trp total** |  | **147.331** |  |  |
| Tyrosine (Tyr) | -554.556305 | Template amino acid |  |  |
| EBT-Tyr (1) | -2411.964316 | 31.742 | 2.31 | -0.389 |
| EBT-Tyr (2) | -2411.957141 | 50.580 | 2.48 | -0.315 |
| EBT-Tyr (3) | -2411.965785 | 27.885 | 2.72 | -0.290 |
| EBT-Tyr (4) | -2411.962481 | 36.561 | 2.52 | -0.364 |
| **19. EBT-Tyr total** |  | **146.768** |  |  |
| Valine (Val) | -323.216119 | Template amino acid |  |  |
| EBT-Val (1) | -2180.629608 | 17.361 | 3.11 | -0.301 |
| EBT-Val (2) | -2180.630982 | 13.752 | 2.87 | -0.251 |
| EBT-Val (3) | -2180.628172 | 21.131 | 3.07 | -0.272 |
| EBT-Val (4) | -2180.626796 | 24.744 | 3.42 | -0.280 |
| **20. EBT-Val total** |  | **76.988** |  |  |


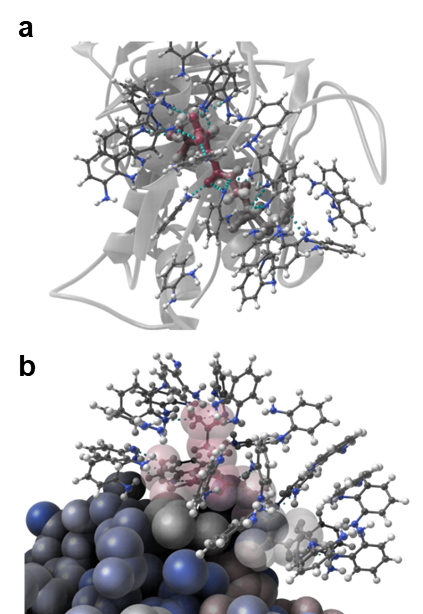


**Figure S12**. Molecular interaction modeling of the o-PD/MMP-8 prepolymerization complex. a) Schematic representation of hydrogen bonding interactions between o-PD monomers and a specific MMP-8 epitope sequence (Ser152, Gln153, Gly154, and Glu155). b) ESP-based schematic illustration of the prepolymerization complex formed between o-PD and the MMP-8 epitope, mapped onto the van der Waals surface, highlighting spatial complementarity and binding affinity.

**Table S3**. Characteristic FT-ATR spectral bands of MIP electrode.

| **Structure** | **Mode** | **Position (cm^-1^)** | **MIP**  **(area)** | **MIP (extract MMP-8) (area)** | **NIP**  **(area)** |
| --- | --- | --- | --- | --- | --- |
| Phenazine | C=N stretching | 1702 | 42.85 | 55.15 | 76.65 |
| Phenazine | Aromatic C=C  stretching | 1518 | 165.59 | 93.48 | 126.78 |
| All area | C-H  methyl rock | 1397 | 65.36 | 83.29 | 97.89 |
| All area | C-N stretching | 1176 | 71.34 | 50.46 | 55.25 |
| Amino acid | -C-OH=C | 998 | 112.32 | -- | -- |
| All area | C-H  out-of-plane | 920 | 105.64 | 47.65 | 52.19 |
| Henazine | N-H, C-H | 600 ~ 800 | N-H bending (wagging, rocking, etc.)  C-H bending, not fully ladder | | |


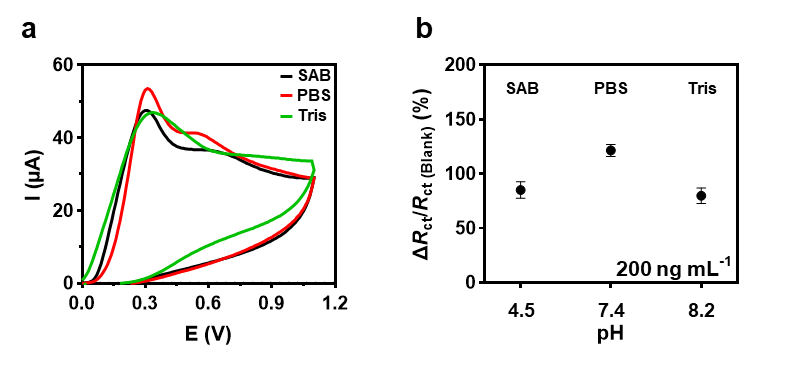


**Figure S13.** Electrochemical evaluation of MIP/GO/SPCE sensor under varying pH conditions. a) Cyclic voltammograms of MIP/GO/SPCE recorded at different pH levels, using SAB, PBS, and Tris buffer solution. b) Comparative electrochemical responses highlighting optimal selectivity and sensitivity at physiological pH 7.4.

**
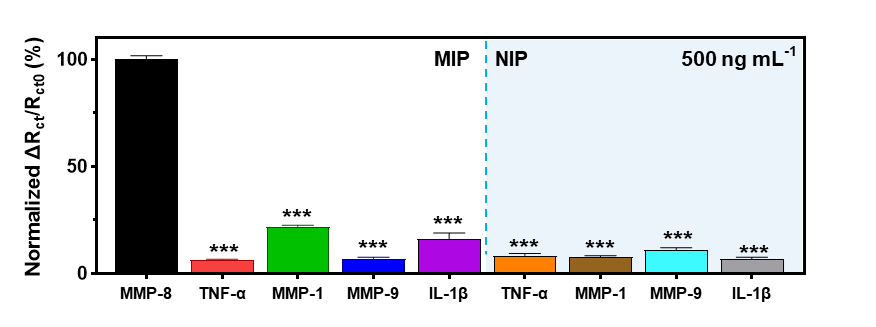
**

**Figure S14.** Selectivity analysis of MIP- and NIP-based sensors against potential interfering cytokines. Normalized Δ*R*_ct/_*R*_ct0_ values demonstrate that the MIP-based electrode exhibits significantly higher impedance responses to MMP-8, compared to TNF-α, MMP-1, MMP-9, and IL-1β. All interferents produced negligible responses in the NIP control. Statistical comparisons (two-tailed unpaired t-test, n ≥ 3) indicate that the signal response to MMP-8 is significantly different from those of all other proteins (^***^*p*< 0.001).


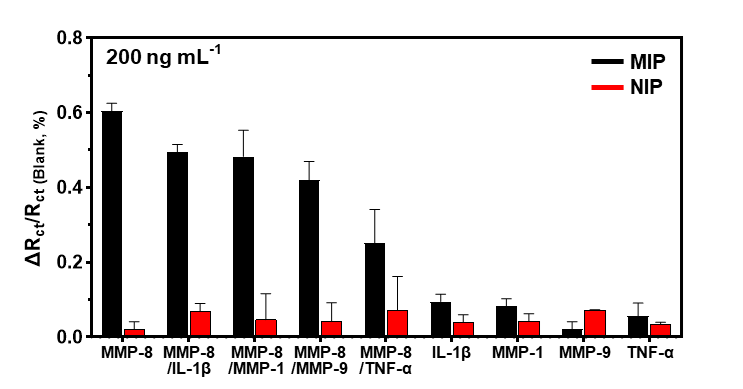


**Figure S15.** Selectivity analysis of MIP-based sensors against potential interferents. Electrochemical responses of the MIP- and NIP-based sensors to the target analyte (i.e., MMP-8) and non-target proteins, including TNF-α, MMP-1, MMP-9, and IL-1β, confirming selective recognition in mixed-protein solutions. The bar graph data correspond to the results presented in Figure 3j.


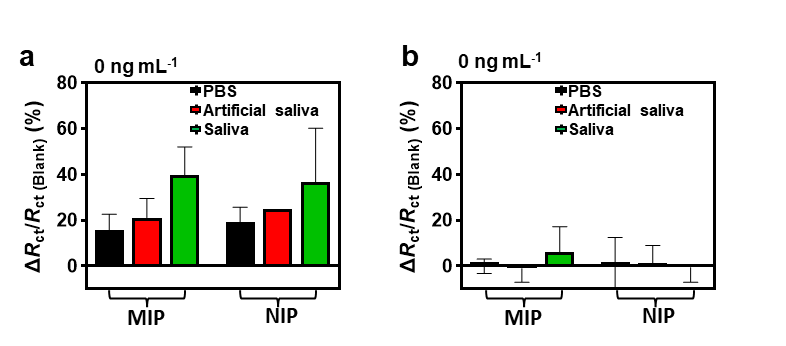


**Figure S16.** Electrochemical responses of MIP- and NIP-functionalized electrodes across different storage conditions and matrices. a) Response of the MIP and NIP electrodes measured immediately after fabrication and final template extraction, following storage in DI water prior to nitrogen drying. b) Electrode performance evaluated after one week of storage under nitrogen, demonstrating preserved functionality.


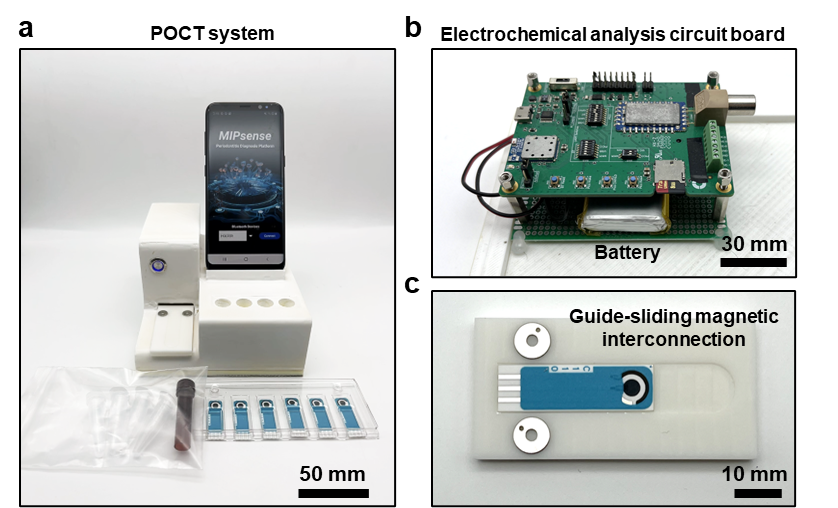


**Figure S17.** Actual digital photographs of the prototype POCT system. a) External design of the 3D-printed, wirelessly enabled POCT device featuring an integrated smartphone holder for user convenience. b) Internal view of the device with the casing removed, showing the built-in battery powering the PCB for electrochemical analysis. c) Close-up of the magnetic sliding guide used for secure insertion of the MIP-based electrode.


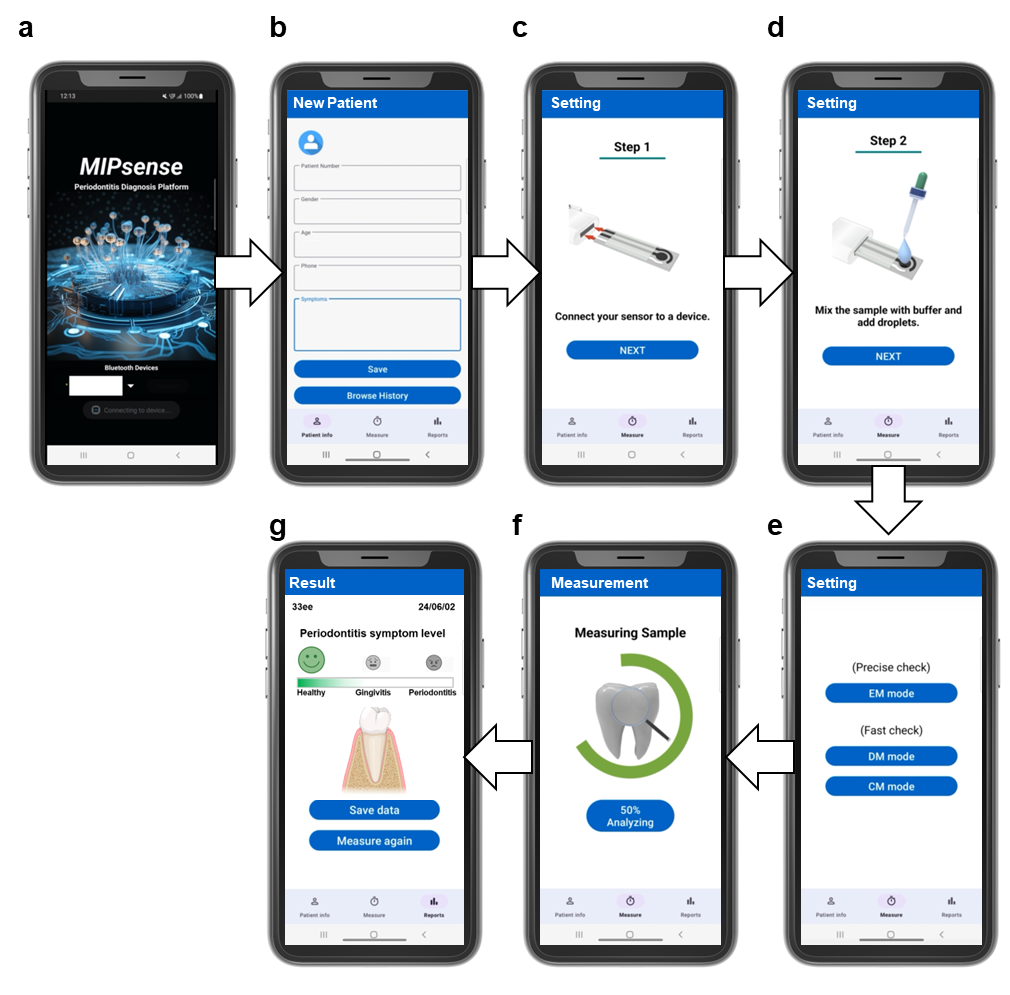


**Figure S18.** Operational overview of the mobile application interface for the smartphone-based POCT system. a) Homepage displaying the main interface of the mobile application. b–g) Stepwise guidance provided by the app for the diagnostic workflow, including: b) input of patient information, c) electrode connection to the device, d) sample application onto the electrode, e) selection of measurement mode, f) feal-time electrochemical measurement, and g) analysis, visualization, and storage of diagnostic results.

**
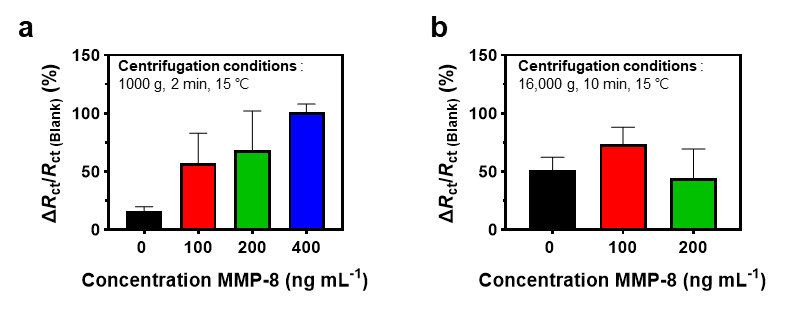
**

**Figure S19.** Sensing responses to standardized MMP-8 concentrations under varying centrifugation conditions with samples collected using the Salivette. a) Stable and concentration-dependent electrochemical responses observed under optimized centrifugation conditions. b) Unstable and inconsistent signals observed under excessive centrifugation, probably due to the co-precipitation of additional salivary components (i.e., small scale contaminants) from the swab, leading to non-specific adsorption on the sensor surface.

**
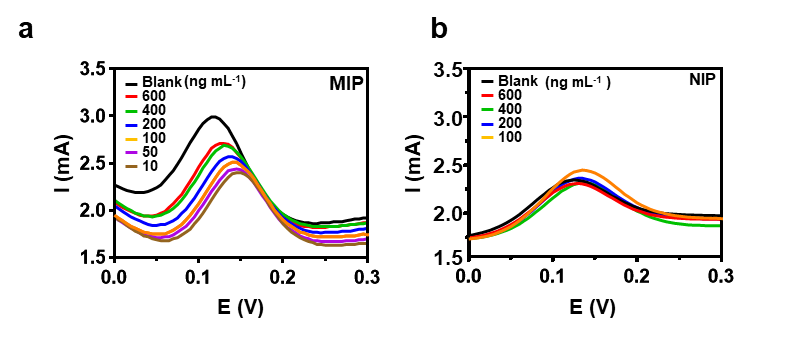
**

**Figure S20.** Characterization and validation of the MIP electrode system. a,b) DPV voltammograms recorded in 0.1 M KCl containing 5 mM K₃[Fe(CN)₆]/K₄[Fe(CN)₆] for MIP and NIP sensors over a concentration range of 10–600 ng mL⁻¹ in artificial saliva.

**
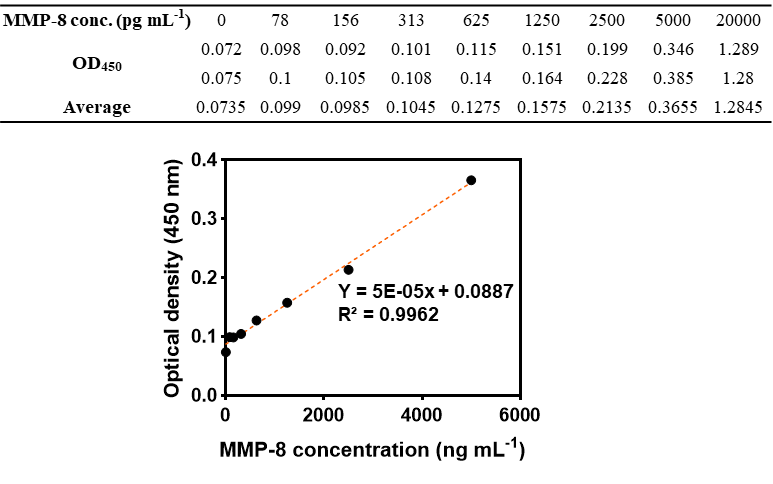
**

**Figure S21.** ELISA standard curve for MMP-8 quantification. MMP-8 concentrations were quantified using a commercial ELISA kit. The standard curve was constructed based on OD_450_ absorbance values measured at various known concentrations of MMP-8. Raw data are tabulated, and background-subtracted mean values (mean ± SD) are plotted in the graph to demonstrate assay linearity and reproducibility.

**Table S4.** Clinical information of periodontitis patients

| **Validation number** | **Sex** | **Age** | **BMI** | **ACH** | **PD** | **PI** | **BOP** |
| --- | --- | --- | --- | --- | --- | --- | --- |
| 1 | M | 34 | 26.4 | 2.717 | 2.685 | 62.5 | 39.743 |
| 2 | F | 40 | 29.76 | 3.94 | 3.92 | 100 | 94.444 |
| 3 | M | 71 | 23.66 | 4.115 | 4.079 | 60.868 | 55.072 |
| 4 | M | 51 | 24.65 | 2.944 | 2.888 | 89.814 | 43.209 |
| 5 | F | 63 | 21.33 | 2.949 | 2.76 | 44.565 | 50.724 |
| 6 | F | 67 | 22.31 | 3 | 2.966 | 52.678 | 50 |
| 7 | F | 65 | 22.51 | 2.761 | 2.648 | 63.392 | 34.523 |
| 8 | M | 67 | 20.7 | 3.121 | 3.015 | 77.272 | 43.939 |
| 9 | F | 48 | 22.32 | 2.59 | 2.537 | 53 | 43.939 |
| 10 | F | 52 | 25.85 | 2.952 | 2.75 | 9.821 | 10.714 |
| 11 | M | 53 | 25.14 | 3.315 | 3.178 | . | 59.523 |
| 12 | F | 71 | 22.86 | 2.944 | 2.888 | 89.814 | 43.209 |
| 13 | F | 48 | 22.94 | 3.273 | 3.273 | 85.714 | 88.095 |
| 14 | M | 55 | 22.14 | 2.928 | 2.809 | 52.678 | 25 |
| 15 | F | 55 | 18.07 | 2.422 | 1.892 | 13.392 | 19.047 |
| 16 | F | 44 | 26.26 | 2.726 | 2.72 | 40 | 32 |
| 17 | F | 49 | 22.76 | 2.773 | 2.773 | 59.821 | 70.238 |
| 18 | F | 68 | 24.52 | 4.177 | 2.666 | 28.225 | 25.806 |
| 19 | F | 64 | 18.67 | 3.04 | 2.66 | 97 | 50.666 |
| 20 | F | 66 | 21.36 | 2.559 | 2.458 | 29.464 | 13.095 |

ACH = alveolar crestal height

PD = probing depth

PI = Plaque index

BOP = bleeding on probing

**Table S5.** Clinical information of healthy patients

| **Validation number** | **Sex** | **Age** | **BMI** | **Ach AL** | **PD** | **PI** | **BOP** |
| --- | --- | --- | --- | --- | --- | --- | --- |
| 1 | M | 26 | 1.93 | 6.222 | 10.5 | 10.5 | 36.48 |
| 2 | M | 24 | 2.241 | 33.87 | 9.375 | 9.375 | 5.92 |
| 3 | F | 25 | 1.97 | 13.392 | 11.428 | 11.428 | 3.87 |
| 4 | M | 31 | 1.76 | 24.137 | 14.137 | 14.137 | 3.62 |
| 5 | M | 25 | 1.964 | 11.904 | 0 | 0 | 3.81 |
| 6 | F | 24 | 1.955 | 0 | 13.46 | 13.46 | 1.73 |
| 7 | M | 24 | 1.517 | 13.392 | 4.761 | 4.761 | 1.55 |
| 8 | M | 24 | 1.833 | 19.354 | 6.451 | 6.451 | 12.97 |
| 9 | F | 28 | 2.255 | 2.38 | 10.714 | 10.714 | 27.97 |
| 10 | F | 27 | 1.392 | 0 | 11.285 | 11.285 | 2.99 |
| 11 | M | 25 | 1.224 | 24.137 | 1.149 | 1.149 | 18.36 |
| 12 | F | 31 | 1.355 | 17.857 | 5.952 | 5.952 | 4.58 |
| 13 | M | 23 | 1.185 | 7.5 | 1.5 | 1.5 | 8.30 |
| 14 | M | 25 | 1.216 | 2.678 | 3.571 | 3.571 | 31.04 |
| 15 | M | 26 | 1.392 | 1.19 | 0.892 | 0.892 | 5.89 |
| 16 | M | 35 | 1.755 | 2.678 | 3.571 | 3.571 | 4.29 |
| 17 | M | 31 | 1.277 | 0 | 8.928 | 8.928 | 9.05 |
| 18 | F | 27 | 1.529 | 2.38 | 7.142 | 7.142 | 6.02 |
| 19 | M | 25 | 1.613 | 8.928 | 0 | 0 | 10.50 |
| 20 | M | 26 | 1.596 | 25.892 | 0 | 0 | 17.20 |

**Table S6.** Clinical information of recovered patient

| **Validation number** | **Sex** | **Age** | **BMI** | **Ach AL** | **Ach PD** | **Ach PI** | **Ach BOP** | **Bch AL** |
| --- | --- | --- | --- | --- | --- | --- | --- | --- |
| 1 | F | 58 | 24.91 | 3.358 | 1.611 | 20.37 | 24.691 | 2.87 |
| 2 | M | 66 | 26.37 | 3.315 | 3.035 | 75 | 47.619 | 1.863 |
| 3 | M | 66 | 22.76 | 5.006 | 2.666 | 73 | 16 | 2.28 |
| 4 | F | 44 | 23.44 | 2.696 | 2.654 | 50 | 22.619 | 2.059 |
| 5 | F | 46 | 19.95 | 2.013 | 1.42 | 42 | 6.666 | 1.926 |
| 6 | F | 50 | 26.69 | 3.057 | 2.988 | 74.137 | 56.321 | 2.35 |
| 7 | M | 56 | 27.78 | 3.48 | 3.005 | 100 | 65.384 | 2.26 |
| 8 | F | 55 | 23.12 | 4.192 | 2.384 | 34.615 | 15.384 | 2.534 |
| 9 | M | 51 | 22.92 | 3.839 | 3 | 15.384 | 26.923 | 2.326 |
| 10 | M | 49 | 24.73 | 3.635 | 3.567 | 59.259 | 65.432 | 2.37 |
| 11 | M | 47 | 23.53 | 3.47 | 2.75 | 54.464 | 39.285 | 1.916 |
| 12 | M | 49 | 24.68 | 3.613 | 2.32 | 4 | 0 | 2.366 |
| 13 | F | 48 | 19.92 | 2.75 | 2.577 | 27.678 | 28.571 | 1.732 |
| 14 | F | 48 | 22.32 | 2.59 | 2.537 | 53 | 43.939 | 1.613 |
| 15 | F | 62 | 19.88 | 2.448 | 2.373 | 12.931 | 3.448 | 1.913 |
| 16 | M | 34 | 26.4 | 2.717 | 2.685 | 62.5 | 39.743 | 2.166 |
| 17 | M | 55 | 22.14 | 2.928 | 2.809 | 52.678 | 25 | 2.255 |
| 18 | F | 53 | 20.58 | 4.086 | 2.895 | 60.185 | 3.703 | 2.425 |
| 19 | M | 48 | 27.73 | 2.937 | 2.723 | 17.968 | 6.25 | 2.484 |
| 20 | M | 50 | 30.74 | 4.722 | 4.388 | 2.083 | 97.222 | 2.976 |

**Table S7.** ELISA results for MMP-8 concentrations in healthy patients.

| **Healthy patient** | **H1** | **H2** | **H3** | **H4** | **H5** | **H6** | **H7** | **H8** | **H9** | **H10** |
| --- | --- | --- | --- | --- | --- | --- | --- | --- | --- | --- |
| **OD_450_** | 0.298 | 0.109 | 0.104 | 0.109 | 0.116 | 0.096 | 0.097 | 0.141 | 0.219 | 0.089 |
| **MMP-8 conc.**  **(pg mL^-1^)** | 4186 | 406 | 306 | 406 | 546 | 146 | 166 | 1046 | 2606 | 6 |
| **X Dilution ratio**  **(×10)** | 41860 | 4060 | 3060 | 4060 | 5460 | 1460 | 1660 | 10460 | 26060 | 60 |
| **MMP-8 conc.**  **(ng mL^-1^)** | 41.86 | 4.06 | 3.06 | 4.06 | 5.46 | 1.46 | 1.66 | 10.46 | 26.06 | 0.06 |
| **Healthy patient** | **H11** | **H12** | **H13** | **H14** | **H15** | **H16** | **H17** | **H18** | **H19** | **H20** |
| **OD_450_** | 0.165 | 0.121 | 0.138 | 0.252 | 0.135 | 0.105 | 0.127 | 0.124 | 0.133 | 0.164 |
| **MMP-8 conc.**  **(pg mL^-1^)** | 1526 | 646 | 986 | 3266 | 926 | 326 | 766 | 706 | 886 | 1506 |
| **X Dilution ratio**  **(×10)** | 15260 | 6460 | 9860 | 32660 | 9260 | 3260 | 7660 | 7060 | 8860 | 15060 |
| **MMP-8 conc.**  **(ng mL^-1^)** | 15.26 | 6.46 | 9.86 | 32.66 | 9.26 | 3.26 | 7.66 | 7.06 | 8.86 | 15.06 |

**Table S8.** ELISA results for MMP-8 concentrations in periodontitis patient

| **Periodontitis patient** | **P1** | **P2** | **P3** | **P4** | **P5** | **P6** | **P7** | **P8** | **P9** | **P10** |
| --- | --- | --- | --- | --- | --- | --- | --- | --- | --- | --- |
| **OD_450_** | 0.0840 | 4.5428 | 0.7650 | 1.0935 | 1.6710 | 1.9721 | 2.0891 | 5.1260 | 0.0769 | 2.6432 |
| **MMP-8 conc. (pg mL^-1^)** | 270.75 | 22564.75 | 3676 | 5318.25 | 8206 | 9711.5 | 10296.25 | 25480.75 | 235.25 | 13066.75 |
| **X Dilution ratio**  **(×10)** | 2707.5 | 225647.5 | 36760 | 53182.5 | 82060 | 97115 | 102962.5 | 254807.5 | 2352.5 | 130667.5 |
| **MMP-8 conc. (ng mL^-1^)** | 2.708 | 225.648 | 36.760 | 53.183 | 82.060 | 97.115 | 102.963 | 254.808 | 2.353 | 130.668 |
| **Periodontitis patient** | **P11** | **P12** | **P13** | **P14** | **P15** | **P16** | **P17** | **P18** | **P19** | **P20** |
| **OD_450_** | 2.2919 | 4.3944 | 4.8622 | 0.2025 | 5.7573 | 0.1029 | 2.0109 | 0.4974 | 0.2016 | 5.9523 |
| **MMP-8 conc. (pg mL^-1^)** | 11310.25 | 21822.75 | 24161.75 | 863.25 | 28637.25 | 365.25 | 9905.5 | 2338 | 859 | 29612.5 |
| **X Dilution ratio**  **(×10)** | 113102.5 | 218227.5 | 241617.5 | 8632.5 | 286372.5 | 3652.5 | 99055 | 23380 | 8590 | 296125 |
| **MMP-8 conc. (ng mL^-1^)** | 113.103 | 218.228 | 241.618 | 8.633 | 286.373 | 3.653 | 99.055 | 23.380 | 8.590 | 296.125 |

**Table S9.** ELISA results for MMP-8 concentrations in recovered patient

| **Recovered patient** | **PP1** | **PP2** | **PP3** | **PP4** | **PP5** | **PP6** | **PP7** | **PP8** | **PP9** | **PP10** |
| --- | --- | --- | --- | --- | --- | --- | --- | --- | --- | --- |
| **OD_450_** | 0.128 | 0.111 | 0.179 | 0.779 | 0.364 | 0.163 | 0.159 | 0.114 | 0.146 | 0.165 |
| **MMP-8 conc. (pg mL^-1^)** | 786 | 446 | 1806 | 13806 | 5506 | 1486 | 1406 | 506 | 1146 | 1526 |
| **X Dilution ratio**  **(×10)** | 7860 | 4460 | 18060 | 138060 | 55060 | 14860 | 14060 | 5060 | 11460 | 15260 |
| **MMP-8 conc. (ng mL^-1^)** | 7.86 | 4.46 | 18.06 | 138.06 | 55.06 | 14.86 | 14.06 | 5.06 | 11.46 | 15.26 |
| **Recovered patient** | **PP11** | **PP12** | **PP13** | **PP14** | **PP15** | **PP16** | **PP17** | **PP18** | **PP19** | **PP20** |
| **OD_450_** | 1.006 | 0.159 | 0.101 | 0.1 | 0.101 | 0.096 | 0.746 | 0.357 | 0.115 | 0.098 |
| **MMP-8 conc. (pg mL^-1^)** | 18346 | 1406 | 246 | 226 | 246 | 146 | 13146 | 5366 | 526 | 186 |
| **X Dilution ratio**  **(×10)** | 183460 | 14060 | 2460 | 2260 | 2460 | 1460 | 131460 | 53660 | 5260 | 1860 |
| **MMP-8 conc. (ng mL^-1^)** | 183.46 | 14.06 | 2.46 | 2.26 | 2.46 | 1.46 | 131.46 | 53.66 | 5.26 | 1.86 |

**Table S10.** MMP-8 concentration profiles across patient groups.

|  | **Periodontitis patient** | | **Healthy patient** | | **Recovered patient** | |
| --- | --- | --- | --- | --- | --- | --- |
| **Validation number** | **Sex** | **ng mL^-1^** | **Sex** | **ng mL^-1^** | **Sex** | **ng mL^-1^** |
| 1 | M | 2.71 | M | 41.86 | F | 7.86 |
| 2 | M | 225.65 | F | 4.06 | M | 4.46 |
| 3 | F | 36.76 | M | 3.06 | M | 18.06 |
| 4 | M | 53.18 | M | 4.06 | F | 138.06 |
| 5 | M | 82.06 | F | 5.46 | F | 55.06 |
| 6 | F | 97.12 | F | 1.46 | F | 14.86 |
| 7 | M | 102.96 | F | 1.66 | M | 14.06 |
| 8 | M | 254.81 | M | 10.46 | F | 5.06 |
| 9 | F | 2.35 | F | 26.06 | M | 11.46 |
| 10 | F | 130.67 | F | 0.06 | M | 15.26 |
| 11 | M | 113.10 | M | 15.26 | M | 183.46 |
| 12 | F | 218.23 | F | 6.46 | M | 14.06 |
| 13 | M | 241.62 | F | 9.86 | F | 2.46 |
| 14 | M | 8.63 | M | 32.66 | F | 2.26 |
| 15 | M | 286.37 | F | 9.26 | F | 2.46 |
| 16 | M | 3.65 | F | 3.26 | M | 1.46 |
| 17 | M | 99.06 | F | 7.66 | M | 131.46 |
| 18 | F | 23.38 | F | 7.06 | F | 53.66 |
| 19 | M | 8.59 | F | 8.86 | M | 5.26 |
| 20 | F | 296.13 | F | 15.06 | M | 1.86 |
| Average |  | 114.4 |  | 11 |  | 34 |
| STDEV |  | 23.0 |  | 11.0 |  | 53.3 |
| n |  | 20 |  | 20 |  | 20 |

**
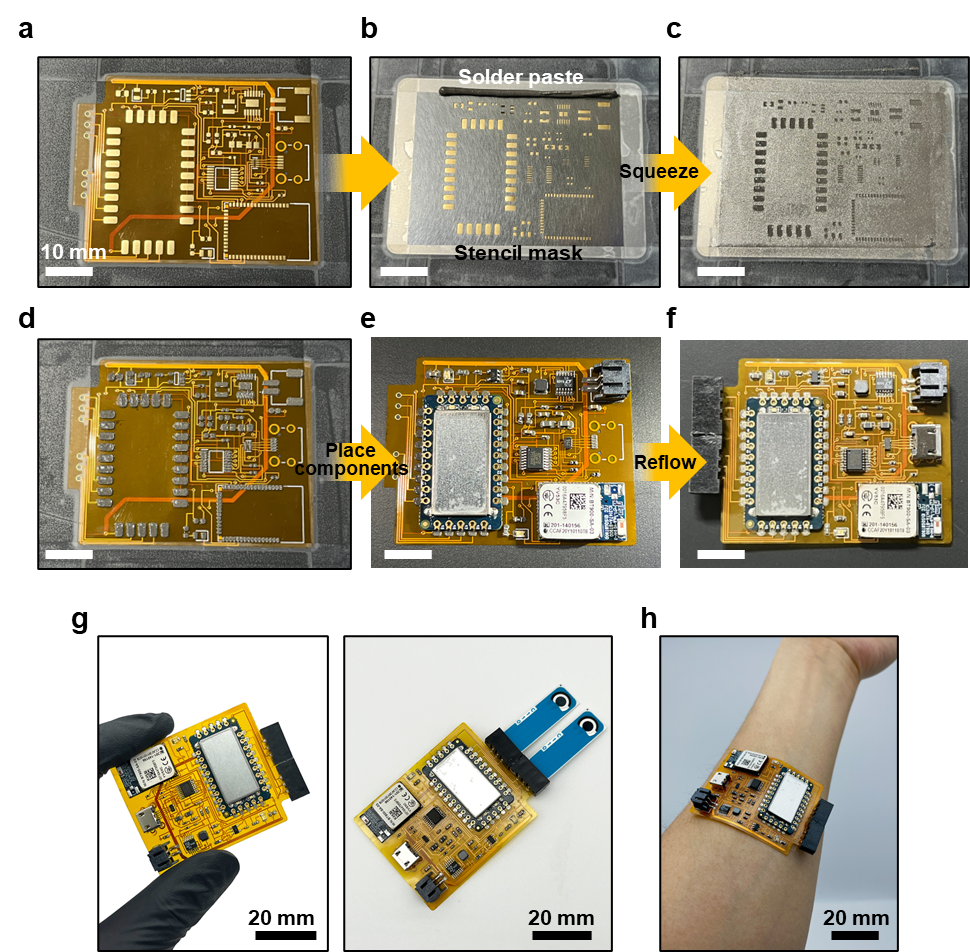
**

**Figure S22.** Light weight and flexible electrochemical analysis device and wireless data transmission. a–f) Stepwise surface mount technology (SMT) process on the FPCB substrate: a) fixation of the FPCB substrate onto a carrier plate, b) alignment of the stencil mask, c) Solder paste deposition using a squeegee blade, d) stencil mask removal, e) component placement, and f) reflow soldering process. g) Digital images of the FPCB-integrated electrochemical analysis device (left) configured with a MIP electrode (right). h) Representative images of a wireless, skin-mountable FPCB-based wearable device, demonstrating mechanical flexibility and practical applicability for epidermal diagnostics for other extended applications.


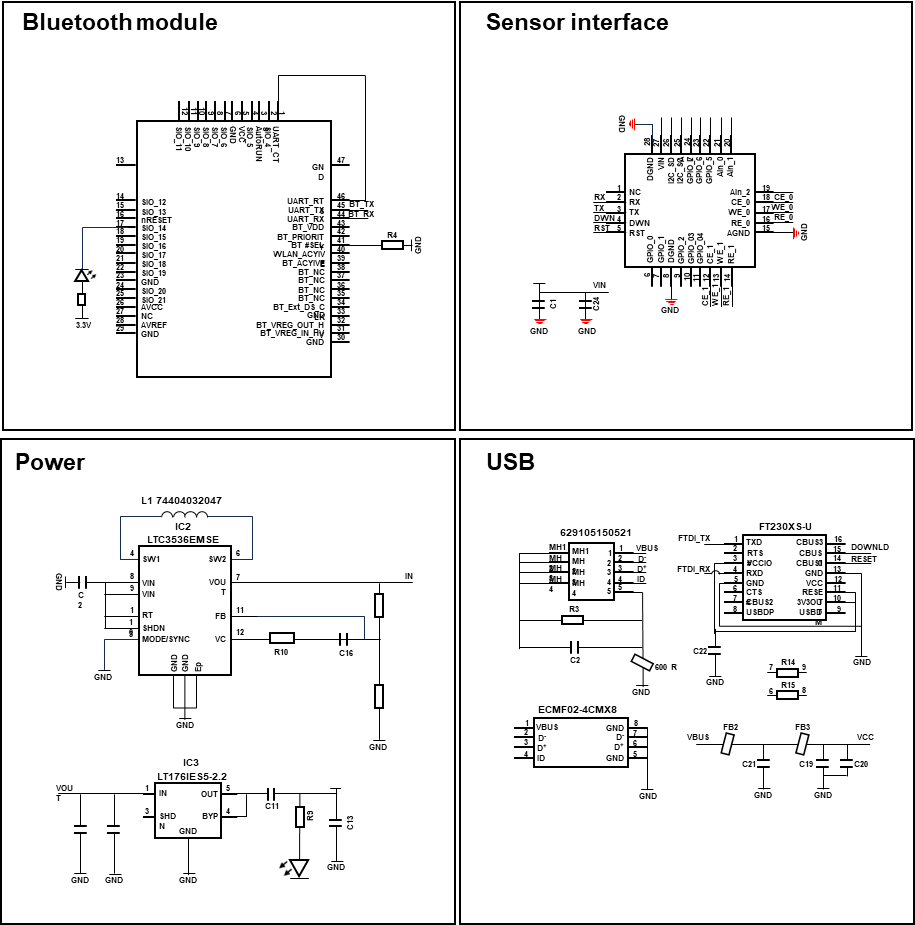


**Figure S23.** Circuit diagram of key components mounted via SMT on the FPCB substrate, as fabricated in Figure S22. SMT Components; Bluetooth (BT-S900), Sensor interface (ADuCM355), Power (L1 74404032047, LTC3536EMSE, LT176IES5-2.2), USB (629105150521, FT230XS-U, ECMF02-4CMX8).


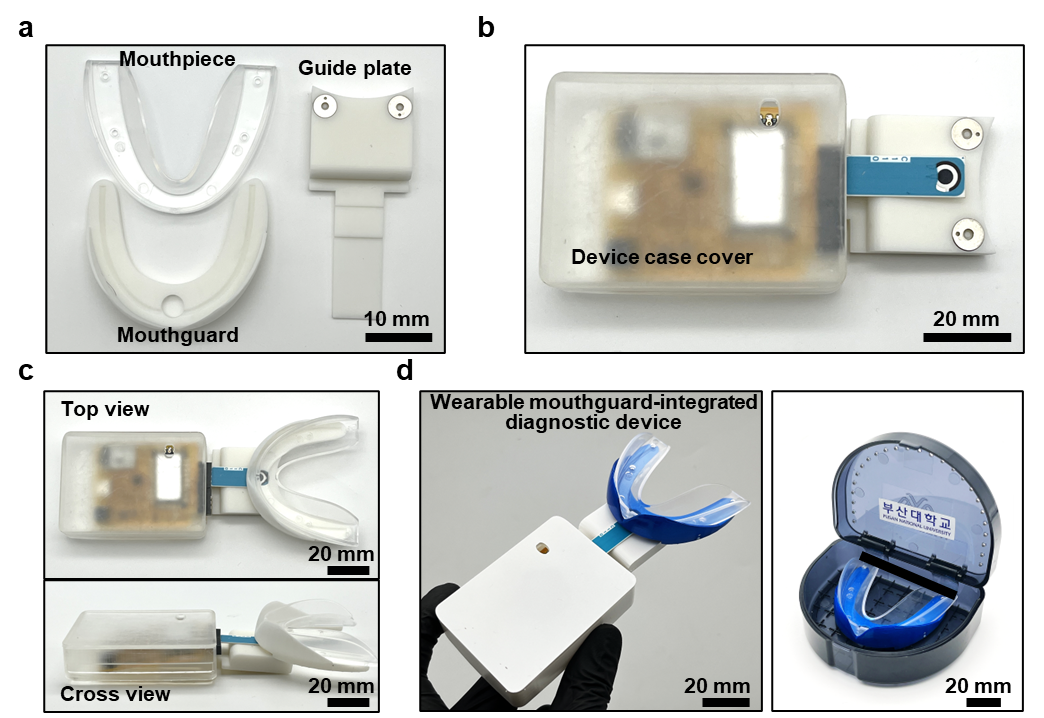


**Figure S24.** Actual images of the wearable device integrated with the mouthguard/mouthpiece assembly presented in Figure 4k. a) Individual hardware components prior to assembly. b) Assembled and enclosed device housing the FPCB-based electrochemical analysis module (refer to Figure S22). c) Final integrated wearable system featuring the embedded MIP-based electrode, combined with the custom-designed mouthguard/mouthpiece and magnetic guide plate for intraoral application. d) Photographs of the prototype MIP electrode-integrated wearable POCT device and its storage usability using a mouthguard case.


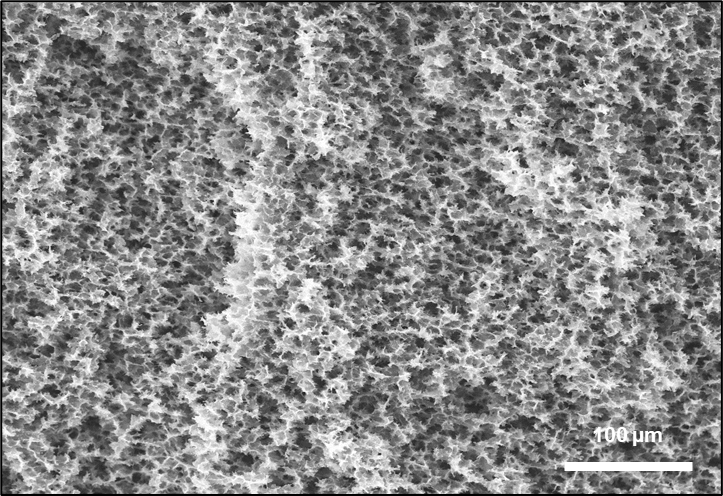


**Figure S25.** SEM image of the PVA-based hydrogel membrane with a highly interconnected porous structure.

**
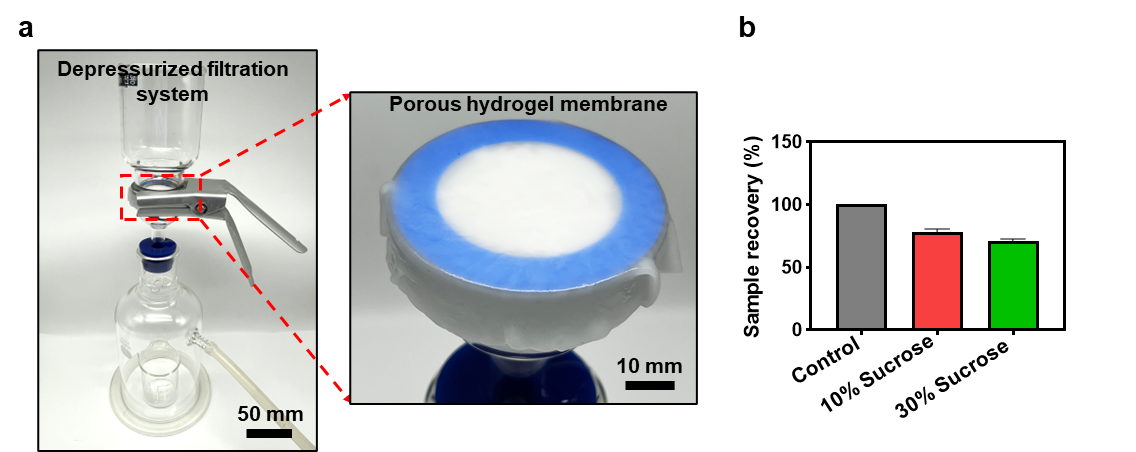
**

**Figure S26.** Depressurized filtration system for protein recovery using a hydrogel membrane. a) Experimental setup for depressurized filtration to evaluate the performance of porous PVA-based hydrogel membranes. b) ELISA-based evaluation of protein recovery efficiency from saliva samples with standardized MMP-8 concentrations, using PVA/KOH membranes formulated with 10% and 30% sucrose. The 10% sucrose membrane (swelling ratio ≈600%) exhibited superior biomolecule retention and stable sensing response, demonstrating an optimal balance between hydrophilicity and molecular permeability.


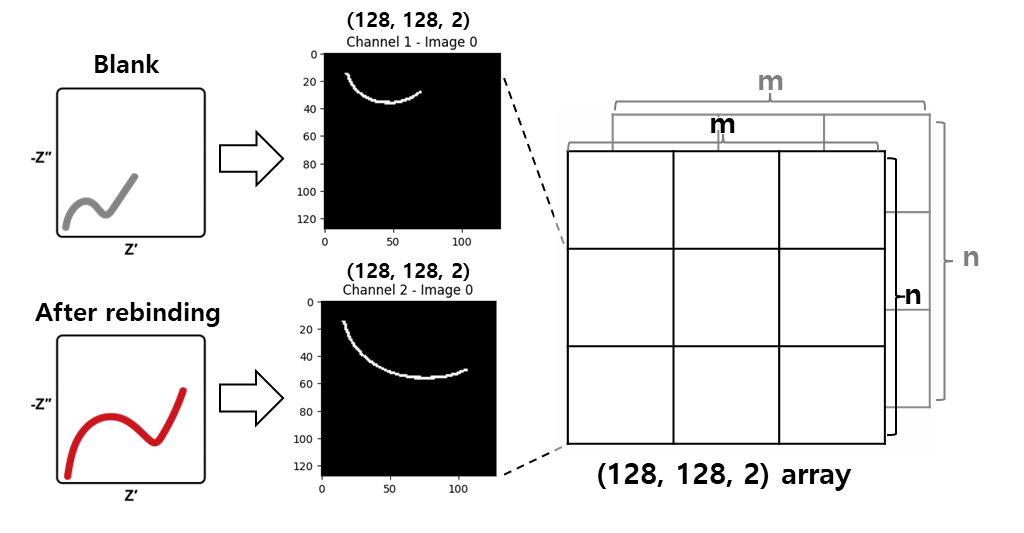


**Figure S27**. Preprocessing pipeline for EIS dataset transformation. The workflow illustrates the conversion of raw numerical EIS data into two-channel binary image representations, preserving critical impedance variations across frequency domains. This transformation enhances feature extraction for subsequent DL-based analysis, which ensures the retention of electrochemical signatures for accurate classification and regression tasks.

**
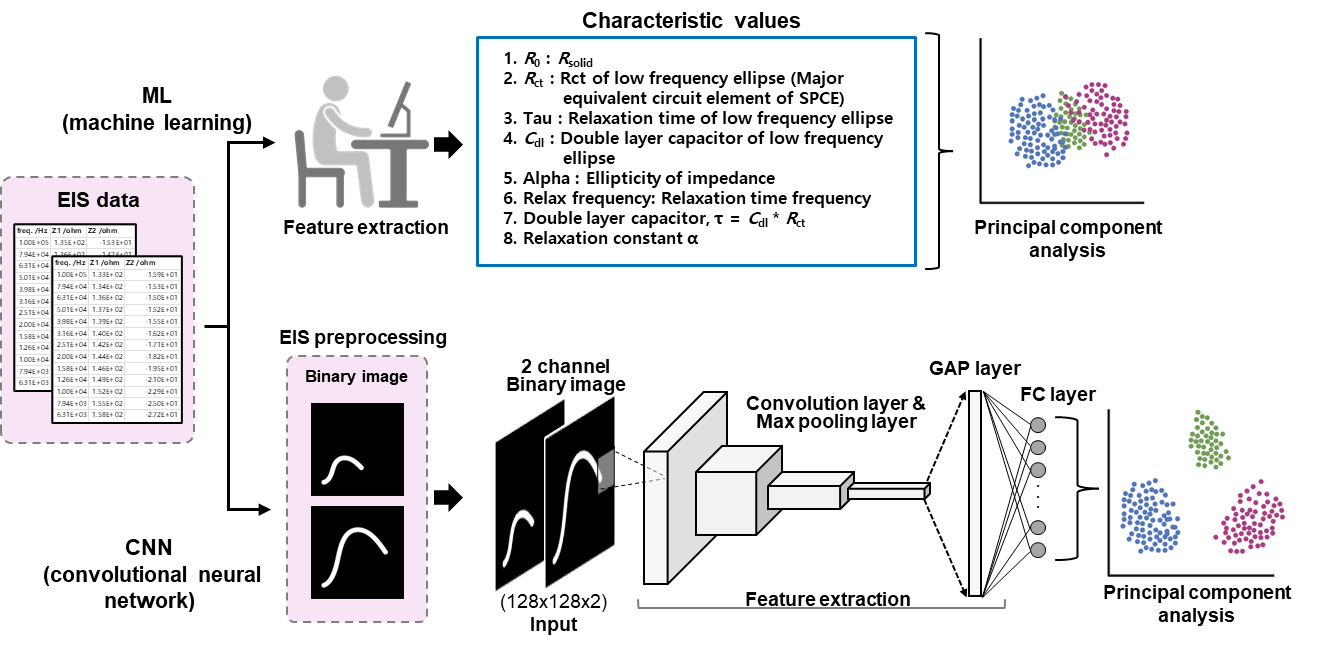
**

**Figure S28.** Comparison of DL-based and conventional ML models for EIS data analysis. The workflow illustrates the conversion of raw EIS numerical data into binary image representations, enabling feature extraction and classification using DL and traditional ML techniques. Exemplified PCA plots visualize the classification performance, highlighting the improved separability achieved through DL-driven feature extraction compared to conventional ML approaches.


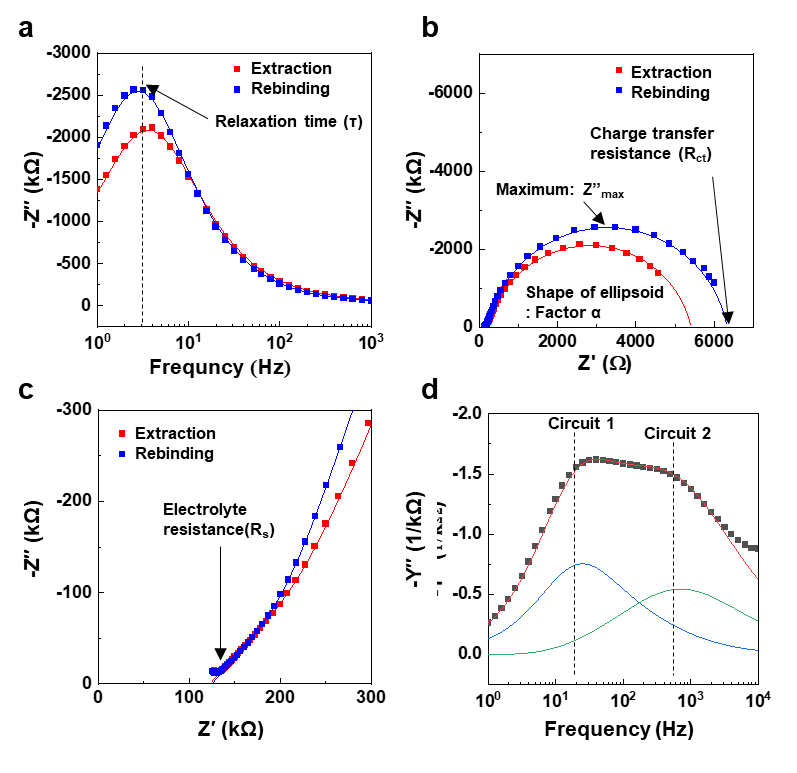


**Figure S29.** a) Frequency-response with fitting curve. b) Nyquist plots by electrochemical impedance spectroscopy for after and before MIP rebinding. c) and zooming graph of Nyquist plots in the high-frequency range. d) Frequency-response with fitting curve by electrochemical admittance (*Y=1/Z*) spectroscopy for MIP electrode.

**Note S1**

To rigorously validate the effectiveness of the DL model, we compared it with classical ML techniques grounded in dielectric relaxation theory, specifically using an equivalent circuit model (ECM). The ECM simplifies complex electrode-electrolyte interfacial interactions into fundamental electrical components. Specifically, the equation (1), (2) will represent the ECM of MIP-based electrode, which simplifies complex electrode-electrolyte interfacial characteristics into electrical components, such as resistors, capacitors, and inductors, connected in series or parallel configurations

| $Z'=R_{s}+R_{ct}\frac{1+\left( w\tau\right)^{\alpha}sin(\frac{1-\alpha}{2}\pi)}{1+\left( w\tau\right)^{2\alpha}+{2\left( w\tau\right)}^{\alpha}sin(\frac{1-\alpha}{2}\pi)}+R_{2}\frac{1+\left( w'\tau' \right)^{\alpha'}sin(\frac{1-\alpha'}{2}\pi)}{1+\left( w'\tau' \right)^{2\alpha'}+{2\left( w'\tau' \right)}^{\alpha'}sin(\frac{1-\alpha'}{2}\pi)}$ | (1) |
| --- | --- |

| $Z"=R_{ct}\frac{\left( w\tau\right)^{\alpha}cos(\frac{1-\alpha}{2}\pi)}{1+\left( w\tau\right)^{2\alpha}+{2\left( w\tau\right)}^{\alpha}sin(\frac{1-\alpha}{2}\pi)}+R_{2}\frac{\left( w'\tau' \right)^{\alpha'}cos(\frac{1-\alpha'}{2}\pi)}{1+\left( w'\tau' \right)^{2\alpha'}+{2\left( w'\tau' \right)}^{\alpha'}sin(\frac{1-\alpha'}{2}\pi)}$ | (2) |
| --- | --- |

Additionally, EIS raw data shows frequency dependence in the frequency response of the imaginary and real parts of the electrical signal. The peak of the curve here represents the relaxation time (i.e. *τ* = 1/2*ωf*) of the ECM of MIP-based electrode. Figures S29a-c display the fitting curves using equations (1) and (2) and the original data simultaneously, which allows the researcher to calculate seven characterization variables: *R*_ct_, τ, α, *R*_s_, *C*_dl_, *f*_τ_. Figure S29d shows the second term of the ECM of MIP-based electrode, which is not clearly visible in the Nyquist plots. This change in representation allowed the researchers to perform more precise fitting.

**Table** **S11**. Feature extraction from EIS data using the ECM for ML-based analysis.

| **No.** | **Type** | **Δ*R*_ct_/*R*_ct0_** | **Δ*τ* /*τ*_0_** | ***α*_0_** | **Δ*α*/*α*_0_** | **Δ*R*_s_/*R*_s0_** | **Δ*C*_dl_/*C*_dl0_** | ***f*/*f*_0_** |
| --- | --- | --- | --- | --- | --- | --- | --- | --- |
| 1 | MIP0 | 0.0669 | 1.2424 | 0.8200 | 0.0305 | -0.0468 | 0.1645 | 1.0000 |
| 2 | MIP0 | 0.0801 | 1.0000 | 0.8000 | 0.0500 | -0.0060 | -0.0741 | 1.2590 |
| 3 | MIP0 | -0.0141 | 1.0753 | 0.8400 | 0.0000 | -0.0794 | 0.0907 | 1.0000 |
| 4 | MIP0 | 0.0445 | 1.0000 | 0.8500 | 0.0000 | 0.0373 | -0.0426 | 1.0000 |
| 5 | MIP0 | 0.0396 | 0.5000 | 0.8800 | 0.0000 | 0.1205 | -0.5190 | 1.9962 |
| 6 | MIP0 | 0.0743 | 1.0000 | 0.9300 | -0.0430 | 0.0755 | -0.0692 | 0.7940 |
| 7 | MIP0 | 0.0722 | 1.1714 | 0.8500 | -0.0471 | 0.0165 | 0.0926 | 1.0000 |
| 8 | MIP0 | 0.0179 | 1.4146 | 0.8400 | 0.0000 | 0.1047 | 0.3898 | 0.6308 |
| 9 | MIP0 | 0.0061 | 0.7531 | 0.8700 | 0.0115 | -0.0833 | -0.2514 | 1.2586 |
| 10 | MIP0 | 0.1060 | 0.8378 | 0.8700 | -0.0345 | 0.0052 | -0.2424 | 1.2588 |
| 11 | MIP0 | 0.1748 | 1.3333 | 0.8600 | -0.0349 | 0.0719 | 0.1349 | 0.7943 |
| 12 | MIP0 | 0.0936 | 1.1277 | 0.8300 | 0.0361 | 0.0316 | 0.0311 | 1.0000 |
| 13 | MIP0 | 0.0626 | 0.9180 | 0.8400 | 0.0000 | 0.0121 | -0.1360 | 1.0000 |
| 14 | MIP0 | 0.0513 | 1.1071 | 0.8300 | 0.0120 | -0.0061 | 0.0531 | 1.0000 |
| 15 | MIP0 | -0.0168 | 1.1622 | 0.8600 | 0.0233 | 0.0084 | 0.1821 | 0.7942 |
| 16 | MIP0 | 0.2190 | 1.4103 | 0.8300 | 0.0000 | -0.0534 | 0.1569 | 1.0000 |
| 17 | MIP0 | 0.1530 | 1.2759 | 0.9200 | -0.0109 | -0.0236 | 0.1065 | 0.7944 |
| 18 | MIP0 | 0.1378 | 1.3103 | 0.9100 | 0.0110 | -0.0238 | 0.1517 | 0.7944 |
| 19 | MIP0 | 0.0914 | 1.1905 | 0.8600 | 0.0233 | -0.0233 | 0.0908 | 0.7942 |
| 20 | MIP0 | 0.1256 | 1.9667 | 0.7500 | 0.1067 | 0.0763 | 0.7472 | 0.6310 |
| 21 | MIP0 | 0.1574 | 1.3056 | 0.8600 | 0.0000 | 0.0328 | 0.1280 | 0.7943 |
| 22 | MIP0 | -0.0168 | 1.1622 | 0.8600 | 0.0233 | 0.0084 | 0.1821 | 0.7944 |
| 23 | MIP0 | 0.2190 | 1.4103 | 0.8300 | 0.0000 | 0.0081 | 0.1569 | 0.7942 |
| 24 | MIP0 | 0.0947 | 1.2653 | 0.8600 | 0.0116 | -0.0159 | 0.1559 | 0.7942 |
| 25 | MIP0 | 0.0949 | 1.0400 | 0.8800 | 0.0114 | -0.0305 | -0.0502 | 1.0000 |
| 26 | MIP0 | 0.2584 | 1.5556 | 0.7700 | 0.0519 | 0.0242 | 0.2361 | 0.6311 |
| 27 | MIP0 | 0.2855 | 1.6522 | 0.8000 | 0.0625 | 0.0000 | 0.2852 | 0.6311 |
| 28 | MIP0 | 0.1118 | 1.1852 | 0.8800 | 0.0227 | 0.0250 | 0.0660 | 0.7944 |
| 29 | MIP0 | 0.1810 | 1.2857 | 0.8700 | 0.0230 | -0.0391 | 0.0887 | 0.7944 |
| 30 | MIP0 | 0.2837 | 1.3462 | 0.8800 | 0.0000 | 0.0000 | 0.0487 | 0.7944 |
| 31 | MIP0 | 0.1415 | 1.3209 | 0.9100 | 0.0160 | 0.0031 | 0.0204 | 0.7943 |
| 32 | MIP0 | 0.1283 | 1.3046 | 0.8600 | 0.0320 | -0.0380 | 0.0400 | 1.0000 |
| 33 | MIP0 | 0.0552 | 1.1622 | 0.8000 | 0.0000 | 0.0610 | -0.0401 | 1.0000 |
| 34 | MIP0 | 0.1299 | 1.4103 | 0.8800 | 0.0116 | 0.0062 | 0.0235 | 0.7942 |
| 35 | MIP0 | 0.2511 | 1.2192 | 0.8700 | 0.0114 | 0.0373 | -0.0426 | 0.7942 |
| 36 | MIP0 | 0.2597 | 1.065 | 0.8600 | 0.0519 | -0.0132 | 0.0450 | 1.0000 |
| 37 | MIP0 | 0.3284 | 1.161 | 0.8300 | 0.0210 | 0.0009 | 0.1009 | 1.2588 |
| 38 | MIP0 | 0.2498 | 1.024 | 0.8400 | 0.0270 | -0.0031 | 0.0932 | 1.0000 |
| 39 | MIP0 | 0.4001 | 1.026 | 0.8700 | 0.0100 | -0.0037 | 0.0716 | 0.6311 |
| 40 | MIP0 | 0.3987 | 1.197 | 0.8800 | 0.0260 | -0.0087 | 0.0751 | 1.0000 |
| 41 | MIP100 | 0.3605 | 1.086 | 0.8800 | 0.0040 | -0.0021 | -0.113 | 0.7942 |
| 42 | MIP100 | 0.4511 | 1.040 | 0.8400 | 0.0280 | 0.1422 | 0.049 | 1.0000 |
| 43 | MIP100 | 0.5849 | 1.033 | 0.8400 | 0.0330 | -0.0377 | -0.059 | 0.6311 |
| 44 | MIP100 | 0.3645 | 1.036 | 0.8700 | 0.0090 | -0.1256 | -0.177 | 1.0000 |
| 45 | MIP100 | 0.7638 | 1.076 | 0.8700 | 0.0300 | -0.1024 | 0.171 | 0.7942 |
| 46 | MIP100 | 0.2833 | 1.073 | 0.8400 | 0.0120 | -0.1143 | -0.079 | 1.0000 |
| 47 | MIP100 | 0.3210 | 1.188 | 0.8400 | 0.0280 | 0.0301 | 0.145 | 0.7944 |
| 48 | MIP100 | 0.4025 | 1.065 | 0.8700 | 0.0210 | 0.1133 | 0.149 | 0.7944 |
| 49 | MIP100 | 0.2379 | 1.180 | 0.8700 | 0.0270 | -0.1374 | 0.139 | 0.7942 |
| 50 | MIP100 | 0.2062 | 1.060 | 0.8800 | 0.0100 | 0.0282 | 0.055 | 0.6310 |
| 51 | MIP100 | 0.2108 | 1.061 | 0.8700 | 0.0260 | 0.0415 | 0.161 | 0.7943 |
| 52 | MIP100 | 0.8177 | 1.014 | 0.8800 | 0.0020 | -0.0885 | 0.065 | 0.7944 |
| 53 | MIP100 | 0.3171 | 1.026 | 0.9300 | 0.0330 | 0.0762 | -0.067 | 0.7942 |
| 54 | MIP100 | 0.2848 | 1.195 | 0.999 | 0.0280 | -0.0807 | 0.067 | 0.7943 |
| 55 | MIP100 | 0.3625 | 1.177 | 0.8600 | 0.0110 | -0.1436 | -0.119 | 1.0000 |
| 56 | MIP100 | 0.6491 | 1.015 | 0.8700 | 0.0080 | -0.1208 | 0.011 | 1.0000 |
| 57 | MIP100 | 0.1367 | 1.119 | 0.8300 | 0.0020 | -0.0879 | -0.122 | 0.6308 |
| 58 | MIP100 | 0.1106 | 1.143 | 0.8800 | 0.0240 | -0.0491 | -0.066 | 1.2586 |
| 59 | MIP100 | 0.3743 | 1.164 | 0.9300 | 0.0020 | 0.0542 | 0.013 | 1.2588 |
| 60 | MIP100 | 0.2367 | 1.060 | 0.8600 | 0.0080 | -0.0563 | -0.077 | 0.7943 |
| 61 | MIP100 | 0.3182 | 1.008 | 0.8700 | 0.0030 | -0.0284 | -0.053 | 1.0000 |
| 62 | MIP100 | 0.4954 | 1.156 | 0.8600 | 0.0010 | 0.0695 | -0.180 | 0.6310 |
| 63 | MIP100 | 0.4731 | 1.121 | 0.8700 | 0.0290 | -0.1051 | -0.169 | 0.7943 |
| 64 | MIP100 | 0.2744 | 1.122 | 0.8300 | 0.0160 | 0.0411 | 0.014 | 0.6310 |
| 65 | MIP100 | 1.2785 | 1.027 | 0.8500 | 0.0320 | 0.0382 | 0.096 | 1.0000 |
| 66 | MIP100 | 1.0648 | 1.051 | 0.8700 | 0.0000 | 0.0549 | 0.080 | 0.6308 |
| 67 | MIP100 | 0.3369 | 1.047 | 0.8300 | 0.0130 | 0.1218 | 0.114 | 1.2586 |
| 68 | MIP100 | 0.2627 | 1.084 | 0.8500 | 0.0270 | -0.0368 | 0.185 | 0.7942 |
| 69 | MIP100 | 0.4127 | 1.170 | 0.8200 | 0.0080 | -0.0387 | 0.056 | 1.0000 |
| 70 | MIP100 | 0.4093 | 1.023 | 0.8400 | 0.0210 | -0.1404 | 0.152 | 0.7942 |
| 71 | MIP100 | 0.2758 | 1.151 | 0.8600 | 0.0300 | 0.0224 | 0.181 | 1.2586 |
| 72 | MIP100 | 0.3152 | 1.1220 | 0.8700 | 0.0210 | 0.0101 | 0.188 | 1.2588 |
| 73 | MIP100 | 0.2287 | 1.5432 | 0.8800 | 0.0130 | 0.0252 | 0.199 | 0.7943 |
| 74 | MIP100 | 0.2931 | 1.2254 | 0.8800 | 0.0000 | 0.0258 | 0.0513 | 0.7943 |
| 75 | MIP100 | 0.4374 | 1.7200 | 0.8500 | 0.0210 | 0.0832 | 0.0781 | 1.0000 |
| 76 | MIP100 | 0.2472 | 1.6890 | 0.8700 | 0.0000 | -0.0520 | 0.0882 | 1.0000 |
| 77 | MIP100 | 0.3674 | 1.3197 | 0.8700 | 0.0300 | -0.0192 | 0.0911 | 0.6308 |
| 78 | MIP100 | 0.3092 | 1.4297 | 0.8600 | 0.0170 | 0.0100 | 0.0974 | 1.2586 |
| 79 | MIP100 | 0.2889 | 1.4479 | 0.8800 | 0.0180 | 0.0564 | 0.0750 | 1.2588 |
| 80 | MIP100 | 0.2458 | 1.4447 | 0.8700 | 0.0105 | 0.0730 | 0.0213 | 0.6310 |
| 81 | MIP200 | 0.5199 | 1.5499 | 0.8600 | 0.0100 | 0.0224 | 0.0539 | 0.5011 |
| 82 | MIP200 | 0.4777 | 1.3157 | 0.8700 | 0.0231 | 0.0166 | 0.0858 | 0.6310 |
| 83 | MIP200 | 0.3780 | 1.2799 | 0.8600 | 0.0449 | -0.0132 | 0.0450 | 0.6310 |
| 84 | MIP200 | 0.6234 | 2.0101 | 0.8600 | 0.0365 | -0.0380 | 0.2400 | 0.5012 |
| 85 | MIP200 | 0.4111 | 1.2282 | 0.8500 | 0.0449 | 0.0610 | -0.0401 | 0.6308 |
| 86 | MIP200 | 0.3596 | 1.2750 | 0.8600 | 0.0189 | -0.0037 | 0.0716 | 0.7946 |
| 87 | MIP200 | 0.5552 | 1.3899 | 0.8500 | 0.0231 | 0.0100 | -0.0769 | 0.7942 |
| 88 | MIP200 | 0.3596 | 1.2750 | 0.8300 | 0.0219 | -0.0159 | 0.0816 | 0.7946 |
| 89 | MIP200 | 0.2666 | 1.2800 | 0.8700 | 0.0355 | -0.0196 | 0.0813 | 0.6310 |
| 90 | MIP200 | 0.5970 | 1.5472 | 0.8400 | 0.0238 | -0.0647 | 0.0312 | 0.6310 |
| 91 | MIP200 | 0.6881 | 1.7714 | 0.8600 | 0.0476 | -0.1541 | -0.0080 | 0.3980 |
| 92 | MIP200 | 1.3643 | 2.5556 | 0.8400 | 0.0238 | -0.0491 | 0.0809 | 0.5011 |
| 93 | MIP200 | 0.4533 | 1.6304 | 0.8700 | 0.0000 | -0.0215 | 0.1219 | 0.6310 |
| 94 | MIP200 | 0.3066 | 1.4390 | 0.8700 | 0.0115 | 0.0324 | 0.1014 | 0.7943 |
| 95 | MIP200 | 0.4389 | 1.4667 | 0.8700 | 0.0115 | 0.0737 | 0.0193 | 0.6310 |
| 96 | MIP200 | 0.3403 | 1.3429 | 0.8700 | 0.0115 | 0.0667 | 0.0019 | 0.6310 |
| 97 | MIP200 | 0.3233 | 1.2571 | 0.8300 | 0.0120 | -0.0150 | -0.0500 | 0.7944 |
| 98 | MIP200 | 0.2901 | 1.2222 | 0.8500 | 0.0471 | 0.0738 | -0.0526 | 0.6310 |
| 99 | MIP200 | 0.4994 | 1.4419 | 0.8200 | 0.0122 | 0.0585 | -0.0384 | 0.7942 |
| 100 | MIP200 | 0.5970 | 1.5472 | 0.8400 | 0.0238 | 0.0647 | -0.0312 | 0.6310 |
| 101 | MIP200 | 0.5157 | 1.4444 | 0.8900 | 0.0225 | 0.0203 | -0.0470 | 0.6310 |
| 102 | MIP200 | 0.5199 | 1.5882 | 0.8700 | 0.0000 | 0.0234 | 0.0449 | 0.6310 |
| 103 | MIP200 | 0.2596 | 1.3750 | 0.8400 | 0.0119 | -0.0057 | 0.0916 | 0.7946 |
| 104 | MIP200 | 0.3380 | 1.2778 | 0.8600 | 0.0349 | -0.0532 | -0.0450 | 0.6310 |
| 105 | MIP200 | 0.4927 | 1.3846 | 0.8600 | 0.0233 | -0.0649 | -0.0724 | 0.7943 |
| 106 | MIP200 | 0.3668 | 1.3704 | 0.8600 | 0.0233 | 0.0000 | 0.0026 | 0.6310 |
| 107 | MIP200 | 0.4024 | 1.3182 | 0.8600 | 0.0349 | 0.0510 | -0.0601 | 0.6308 |
| 108 | MIP200 | 0.2765 | 1.2174 | 0.8700 | 0.0115 | 0.0000 | -0.0463 | 1.0000 |
| 109 | MIP200 | 0.4744 | 1.4082 | 0.8600 | 0.0349 | -0.0109 | -0.0449 | 0.7942 |
| 110 | MIP200 | 0.5032 | 1.3878 | 0.8700 | 0.0230 | 0.0000 | -0.0768 | 0.7942 |
| 111 | MIP200 | 0.0159 | 1.2000 | 0.8800 | 0.0455 | -0.0296 | 0.1813 | 0.6310 |
| 112 | MIP200 | 0.4274 | 1.4118 | 0.8500 | 0.0000 | 0.0238 | -0.0109 | 0.6310 |
| 113 | MIP200 | 0.2930 | 1.3030 | 0.8800 | 0.0227 | -0.0084 | 0.0078 | 0.7944 |
| 114 | MIP200 | 0.3721 | 1.3115 | 0.9000 | 0.0111 | -0.0427 | 0.2233 | 0.7946 |
| 115 | MIP200 | 0.3886 | 1.0385 | 0.8600 | 0.0116 | -0.0083 | -0.0461 | 1.0000 |
| 116 | MIP200 | 0.6000 | 2.0000 | 0.8700 | 0.0345 | -0.0360 | 0.2500 | 0.5012 |
| 117 | MIP200 | 0.4012 | 1.6842 | 0.8100 | 0.0494 | 0.0000 | 0.2020 | 0.6309 |
| 118 | MIP200 | 0.3691 | 1.7083 | 0.8500 | 0.0353 | -0.0081 | 0.2478 | 0.6311 |
| 119 | MIP200 | 0.4852 | 1.2857 | 0.8700 | 0.0230 | 0.0163 | 0.0848 | 0.6311 |
| 120 | MIP200 | 0.5034 | 1.2581 | 0.8500 | 0.0235 | -0.0077 | 0.1402 | 0.7944 |

**Table S12**. Standard 2 × 2 contingency table of MMP-8 standard samples based on OPLS-DA analysis results.

| **0 & 200 ng mL^-1^** | | **Actual status** | |
| --- | --- | --- | --- |
|  |  | Diagnosis positive | Diagnosis negative |
| Assignment  status | Test positive | Ture positive (TP)  90.9% | False positive (FP)  9.1% |
|  | Test negative | False negative (FN)  11.8% | Ture negative (TN)  88.2% |
|  | Screening | Sensitivity = TP / (TP + FN)  88.5% | Specificity = TN / (TN + FP)  90.7% |
|  |  | Precision = TP / (TP+FP)  90.9% | Accuracy = (TP + TN) / All  89.6% |
| **0 & 100 ng mL^-1^** | | **Actual status** | |
|  |  | Diagnosis positive | Diagnosis negative |
| Assignment  status | Test positive | Ture positive (TP)  72.7% | False positive (FP)  27.3% |
|  | Test negative | False negative (FN)  29.0% | Ture negative (TN)  71.0% |
|  | Screening | Sensitivity = TP / (TP + FN)  71.5% | Specificity = TN / (TN + FP)  72.2% |
|  |  | Precision = TP / (TP+FP)  72.7% | Accuracy = (TP + TN) / All  71.8% |
| **100 & 200 ng mL^-1^** | | **Actual status** | |
|  |  | Diagnosis positive | Diagnosis negative |
| Assignment  status | Test positive | Ture positive (TP)  72.3% | False positive (FP)  38.7% |
|  | Test negative | False negative (FN)  23.5% | Ture negative (TN)  76.5% |
|  | Screening | Sensitivity = TP / (TP + FN)  72.3% | Specificity = TN / (TN + FP)  66.4% |
|  |  | Precision = TP / (TP+FP)  61.3% | Accuracy = (TP + TN) / All  68.9% |

**Table S13**. Confusion Matrices for DL-Based classification of MMP-8 standard samples.

| **0 & 200 ng mL^-1^** | | **Actual status** | |
| --- | --- | --- | --- |
|  |  | Diagnosis positive | Diagnosis negative |
| Assignment  status | Test positive | Ture Positive (TP)  100 % | False Positive (FP)  0 % |
|  | Test negative | False Negative (FN)  0 % | Ture Negative (TN)  100 % |
|  | Screening | Sensitivity = TP / (TP + FN)  100 % | Specificity = TN / (TN + FP)  100 % |
|  |  | Precision = TP / (TP+FP)  100 % | Accuracy = (TP + TN) / All  100 % |
| **0 & 100 ng mL^-1^** | | **Actual status** | |
|  |  | Diagnosis positive | Diagnosis negative |
| Assignment  status | Test positive | Ture Positive (TP)  98.3% | False Positive (FP)  0 % |
|  | Test negative | False Negative (FN)  1.6 % | Ture Negative (TN)  100.0% |
|  | Screening | Sensitivity = TP / (TP + FN)  98.4 % | Specificity = TN / (TN + FP)  100 % |
|  |  | Precision = TP / (TP+FP)  100 % | Accuracy = (TP + TN) / All  99.2 % |
| **100 & 200 ng mL^-1^** | | **Actual status** | |
|  |  | Diagnosis positive | Diagnosis negative |
| Assignment  status | Test positive | Ture Positive (TP)  100 % | False Positive (FP)  0 % |
|  | Test negative | False Negative (FN)  0 % | Ture Negative (TN)  100 % |
|  | Screening | Sensitivity = TP / (TP + FN)  100 % | Specificity = TN / (TN + FP)  100 % |
|  |  | Precision = TP / (TP+FP)  100 % | Accuracy = (TP + TN) / All  100 % |


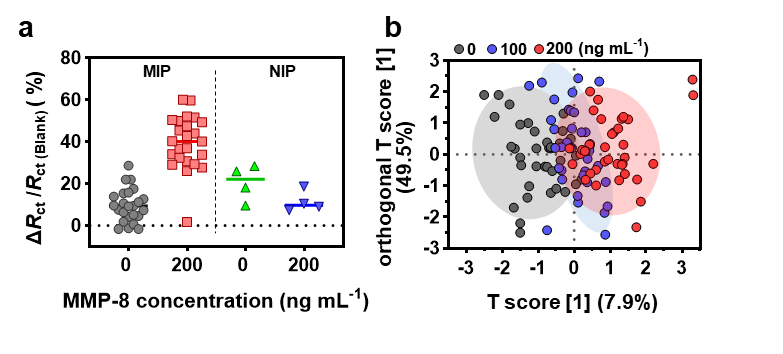


**Figure S30.** Orthogonal partial least squares discriminant analysis (OPLS-DA) score plot for the classification of MMP-8 standard sample tests. The plot illustrates the separation of different MMP-8 concentration groups, with distinct clusters representing variations in molecular recognition. The ellipses denote the confidence interval, demonstrating moderate classification accuracy and variance within the dataset; cluster separation indicates the model’s partial capability to distinguish MMP-8 levels.


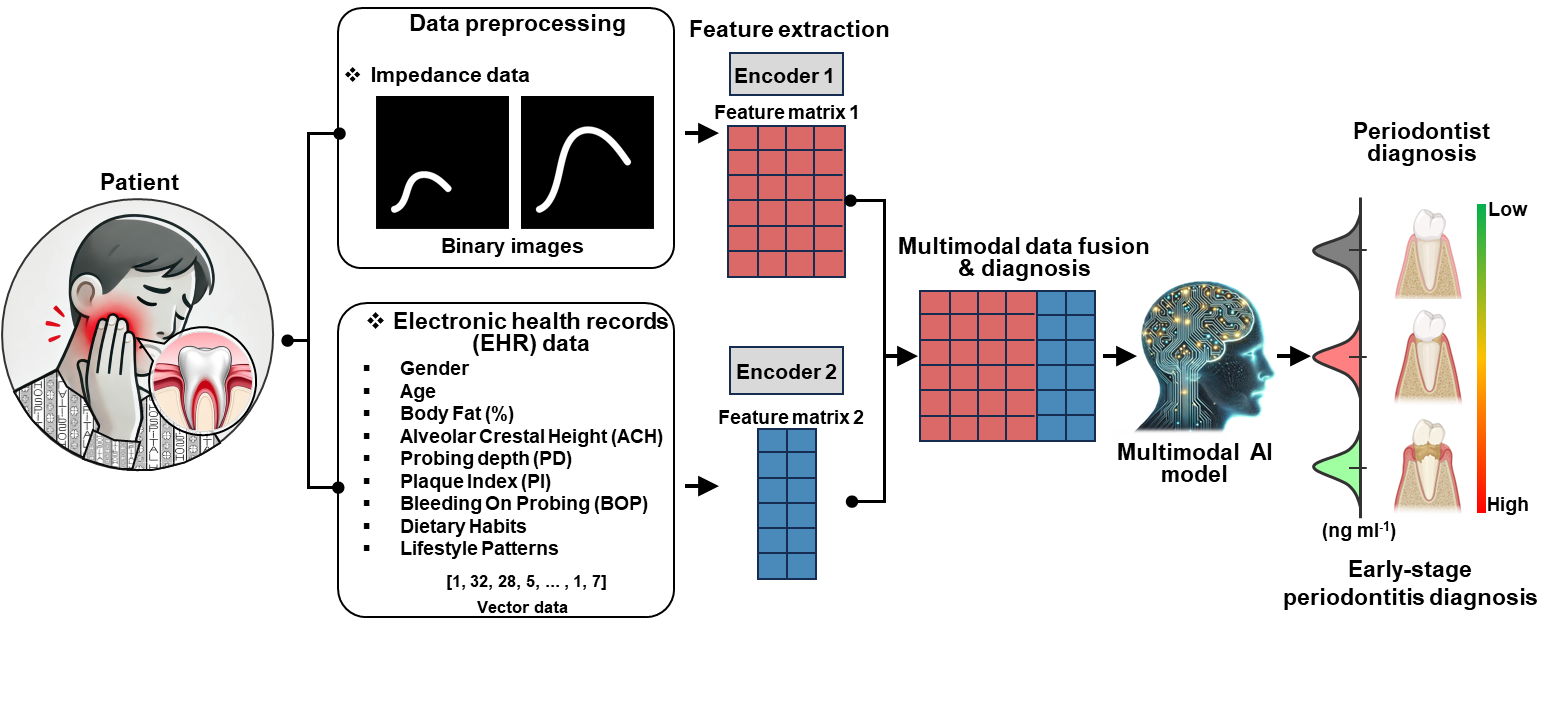


**Patient**

**Figure S31.** Scheme of multimodal DL framework for periodontitis diagnosis. Hybrid model integrating electrochemical data and clinical metadata using multimodal DL algorithms to enhance diagnostic accuracy and reliability for complex multifactorial diseases.


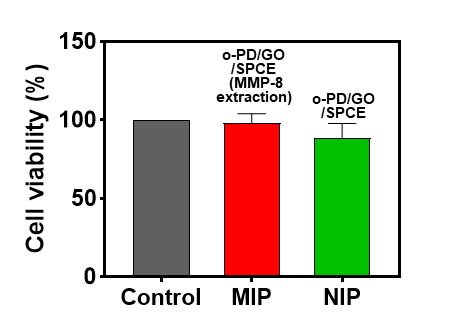


**Figure S32.** In vitro cytocompatibility assessment of the MIP electrode after template extraction. L929 fibroblast cells exhibited high viability on the MIP-modified and NIP electrode, confirming its suitability for intraoral diagnostic applications.
